# *Aspergillus fumigatus* effector crpA orchestrates host prostaglandin signaling to promote fungal virulence

**DOI:** 10.1128/mbio.02481-25

**Published:** 2025-09-12

**Authors:** Camila Figueiredo Pinzan, Camila Diehl, Patrícia Alves de Castro, Endrews Delbaje, Peter Rocha, Camila Langer Marciano, Nathalia Gonsales da Rosa-Garzon, Hamilton Cabral, Rosanne Sprute, Androniki Kolovou, Adriana Ferreira Lopes Vilela, Carlos Arterio Sorgi, Agathe Ecoutin, Mélanie Berbon, Antoine Loquet, Edismauro Garcia Freitas-Filho, Larissa Dias Cunha, Savini U. Thrikawala, Emily E. Rosowski, Thaila Fernanda dos Reis, Gustavo H. Goldman

**Affiliations:** 1 Faculdade de Ciências Farmacêuticas de Ribeirão Preto, Universidade de São Paulo 67782 , Ribeirão Preto, Brazil; 2 Institute of Translational Research, Cologne Excellence Cluster on Cellular Stress Responses in Aging-Associated Diseases (CECAD), Faculty of Medicine and University Hospital Cologne, University of Cologne 506060 , Cologne, Germany; 3 Department I of Internal Medicine, Excellence Center for Medical Mycology (ECMM), Faculty of Medicine and University Hospital Cologne, University of Cologne 61059, Cologne, North Rhine-Westphalia, Germany; 4 German Centre for Infection Research (DZIF), Partner Site Bonn-Cologne, Cologne, Germany; 5 Departamento de Química, Faculdade de Filosofia, Ciências e Letras de Ribeirão Preto, Universidade de São Paulo 124588 , Ribeirão Preto, Brazil; 6 Univ. Bordeaux, CNRS, Bordeaux INP, CBMN, UMR 5248, IECB, Pessac, France; 7 Departamento de Biologia Celular e Molecular e Bioagentes Patogênicos, Faculdade de Medicina de Ribeirão Preto (FMRP), Universidade de São Paulo (USP) 54539 , Ribeirão Preto, Brazil; 8 Department of Biological Sciences, Clemson University 170362, Clemson, South Carolina, USA; 9 Eukaryotic Pathogens Innovation Center, Clemson University 2545, Clemson, South Carolina, USA; 10 National Institute of Science and Technology in Human Pathogenic Fungi, São Paulo, Brazil; Institut Pasteur, Paris, France

**Keywords:** *Aspergillus fumigatus*, prostaglandin, virulence, cytokines, solid-state NMR

## Abstract

Conidia are the primary infection structures in *Aspergillus fumigatus*, the etiologic agent of aspergillosis. Here, we characterized CrpA (a cysteine-rich protein), a conidial surface-associated protein important for fungal evasion and host immunity modulation. Δ*crpA* conidia elicited decreased production of proinflammatory cytokines and increased production of anti-inflammatory cytokine IL-10 from murine macrophages and in the lungs of infected mice. Murine macrophages exposed to Δ*crpA* conidia produce significantly higher levels of prostaglandins PGE2 and PGD2, suggesting that deletion of CrpA modulates cytokine production through effects on eicosanoid signaling. While Δ*crpA* spores have lower virulence in larval zebrafish, this difference is abrogated in larvae that cannot produce prostaglandins. The CrpA protein can directly modulate PGE2 and cytokine production by macrophages, and solid-state NMR shows that Δ*crpA* swollen conidia present lower β−1,3-glucan and chitin than the wild-type strain, suggesting that the effects of the Δ*crpA* mutant on macrophages are due to the combinatorial effects of direct CrpA action and altered cell wall PAMP recognition. Δ*crpA* mutants are avirulent in an immunocompetent murine model of aspergillosis, and high CrpA-specific IgG responses were found in antisera from individual patients with invasive pulmonary aspergillosis, suggesting a role for CrpA in *A. fumigatus* pathogenesis.

**IMPORTANCE:**

Conidia serve as the primary infectious units of *Aspergillus fumigatus*, the causative agent of aspergillosis. This study identifies CrpA, a cysteine-rich protein found on the conidial surface, as a crucial regulator of immune modulation and fungal virulence. Loss of CrpA (Δ*crpA*) alters host immune responses, resulting in reduced production of proinflammatory cytokines and increased IL-10 levels in both murine macrophages and infected lungs. ΔcrpA conidia also stimulate elevated levels of prostaglandins PGE2 and PGD2. This immunomodulatory effect is dependent on eicosanoid signaling as the virulence of Δ*crpA* is restored in prostaglandin-deficient zebrafish larvae. CrpA directly modulates macrophage production of PGE2 and cytokines. Solid-state NMR analysis shows that Δ*crpA* conidia expose lower levels of β−1,3-glucan and chitin, suggesting that CrpA influences both cell wall composition and host pattern recognition receptor engagement. Δ*crpA* strains are avirulent in immunocompetent mice, and patients with invasive pulmonary aspergillosis exhibit elevated CrpA-specific IgG. These results highlight CrpA as a key virulence factor in *A. fumigatus* and a promising target for antifungal therapy.

## INTRODUCTION

*Aspergillus fumigatus* is a saprophytic, thermotolerant fungus that can cause several clinical entities, collectively known as aspergillosis. Humans continuously breathe in hundreds to thousands of *A. fumigatus* conidia each day. However, most immunocompetent individuals can clear these conidia through their innate immune defenses. There are three main types of disease caused by *A. fumigatus: Aspergillus* allergic disease, chronic pulmonary aspergillosis (CPA) in patients with underlying structural lung disease, and the most lethal form, invasive pulmonary aspergillosis (IPA), which affects immunosuppressed patients (1–3). In immunocompromised individuals, failures in the host’s innate immune function contribute to the inability to clear inhaled *A. fumigatus* conidia, which eventually germinate, grow as invasive hyphae, and potentially disseminate. Infections caused by *A. fumigatus* in immunocompromised patients due to malignancy, transplantation, and corticosteroid use have dramatically increased in recent years. IPA can also affect patients with "non-classical" risk factors, such as non-neutropenic patients with respiratory viral infections, chronic obstructive pulmonary disease, cirrhosis, or autoimmune diseases treated with novel biologics. These patients exhibit varying degrees of innate immune competence, which can impact the clearance of conidia and the complete removal of germlings or hyphae, allowing survivors to evade or escape the residual innate immune response.

*A. fumigatus* conidia are the first structures that encounter the host immune system. The conidial cell wall is composed of β−1,3-glucan and chitin that are enveloped by sheets of rodlet and melanin, where proteins are anchored (4, 5). Conidial surface proteins are important for morphogenesis, to combat environmental stressors, substrate adherence, and virulence (6). The hydrophobin RodA is a significant component of the rodlet layer and is essential for cell wall physical resistance and permeability, preventing immune recognition (7, 8). *A. fumigatus* CcpA is essential to maintain the correct surface structure and prevent immune recognition (9). In contrast to fungal plant pathogens, little is known about protein effectors in human fungal pathogens. Recently, a few effector proteins have been identified in human fungal pathogens (10–15).

There is scarce information about *A. fumigatus* proteins involved in fungal evasion and host immunity modulation. *A. fumigatus* HscA has been demonstrated to anchor human p11 on phagosomal compartments in epithelial cells, rewiring the vesicular trafficking to the nondegradative pathway, facilitating the escape of conidia (13). Recently, we analyzed the conidial surface proteome of *A. fumigatus*, two closely related nonpathogenic species, *Aspergillus fischeri* and *Aspergillus oerlinghausenensis*, as well as pathogenic *Aspergillus lentulus*, to identify such proteins (14). After identifying 62 proteins exclusively detected on the *A. fumigatus* conidial surface, we assessed null mutants for 42 genes encoding these proteins. Deleting 33 of these genes altered susceptibility to macrophages, epithelial cells, and cytokine production. Notably, a gene that encodes a putative glycosyl asparaginase (AspA), modulating the host proinflammatory cytokine IL-1β levels, is important for infection in an immunocompetent murine model of fungal disease. In this data set, we have also identified a protein, CrpA (cysteine-rich protein), whose encoding gene was not further characterized. Here, we assessed the influence of *crpA* null and overexpression mutants on host immunity modulation. Wild-type *A. fumigatus* can release free fatty acids, such as omega-3 EPA, and induce the production of several eicosanoids, including 15-oxo-ETE and prostaglandins (PGs) PGE2 and PGD2 by murine macrophages. Interestingly, stimulation with Δ*crpA* spores induces increased production of PGE2, PGD2, and the corresponding metabolized products PGA2/PGJ2 by macrophages. Modulation of prostaglandin signaling affects cytokine production, and Δ*crpA* spores induce decreased macrophage production of proinflammatory cytokines, such as TNF-α, IL-1β, and IL-6, but increased anti-inflammatory IL-10. Δ*crpA* spores are less virulent in neutrophil-defective larval zebrafish, but this decrease is abrogated in *ptgs2* mutant larvae, which cannot produce prostaglandins. We find both that the CrpA protein can have direct effects on cytokine production and that the cell wall of Δ*crpA* mutants is altered, potentially affecting PAMP recognition. Δ*crpA* mutants are avirulent in an immunocompetent murine model but virulent in a chemotherapeutic murine model of aspergillosis, and antibodies against CrpA can be found in IPA patient sera, suggesting that CrpA is a fungal factor affecting disease pathogenesis.

## RESULTS

### CrpA, a cysteine-rich protein from *A. fumigatus*

**Fig 1:**
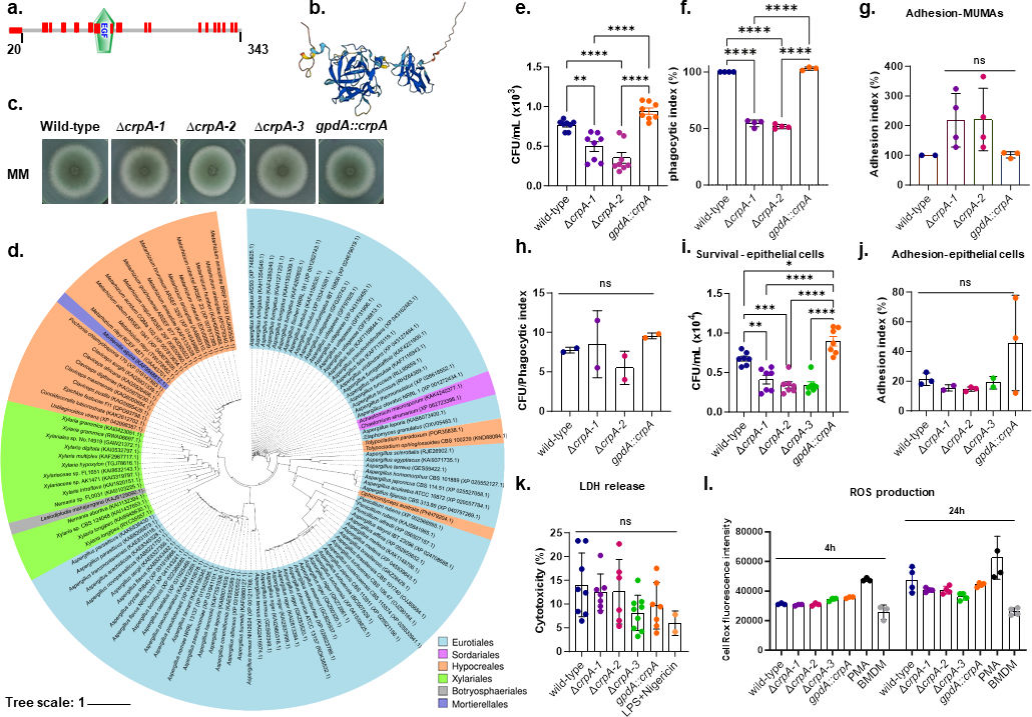
*A. fumigatus* crpA encodes a cysteine-rich protein that has a restricted phylogenetic distribution. (**a**) CrpA protein organization. Cysteine residues are labeled in red, and an epidermal growth factor-like domain (EGF) http://smart.embl-heidelberg.de/smart/job_status.pl?jobid=143107200194240251737545792biaJKFQYmE) is shown. (**b**) Proposed CrpA alpha-fold structure. (**c**) *A. fumigatus* wild-type, Δ*crpA-1*, Δ*crpA-2*, Δ*crpA-3*, and *gpdA::crpA* strains were grown for 3 days at 37°C in MM. (**d**) Phylogenetic distribution of CrpA in fungal classes. (**e**) The Δ*crpA* mutants have reduced viability by MUMAs when compared to the wild-type strain. MUMAs were challenged with *A. fumigatus* conidia at a multiplicity of infection (MOI) of 1:10. After 24  h of incubation, the cell suspensions were seeded, and the number of CFU per mL was evaluated. The statistical analysis was performed using a two-sided one-way ANOVA (Tukey’s test) for multiple comparisons. Data are presented as values from two biological experiments with three replicates ±standard deviation, **P < 0.005; ****P < 0.0001 versus A1163 wild-type strain. (**F and G**) The Δ*crpA* mutants have reduced viability by the MUMAs when compared to the wild-type strain. The phagocytic index was calculated by the number of conidia that had been phagocytosed for each macrophage, and the adhesion index was calculated by the number of conidia counted on each macrophage surface (each point plotted is a cell). Fifty cells were counted for each data set in two independent experiments, totaling 100 cells. (**h**) The Δ*crpA* mutants have comparable viability to the wild-type strain when the CFUs are normalized by the phagocytic index. Statistical analysis was performed using a two-sided one-way ANOVA (Tukey’s test) for multiple comparisons. ***P < 0.0005; ****P < 0.0001; ns = nonsignificant. (**i**) The ΔcrpA mutants have reduced viability in the A549 lung epithelial cells when compared to the wild-type strain. A549 cells were challenged with *A. fumigatus* conidia at an MOI of 1:10. After 24  h of incubation, the cell suspensions were seeded, and the number of CFU per mL was evaluated. (**j**) The Δ*crpA* mutants have comparable adhesion to A549 epithelial cells when compared to the wild-type strain. Statistical analysis was performed using a two-sided one-way ANOVA (Tukey’s test) for multiple comparisons. Adjusted (adj.) *P* values. *adj. 0.0186; **adj. 0.0032; ***adj. 0.0002 and ****P < 0.0001. (**k**) Viability of MUMAs exposed to 24 h at 37°C to the wild-type and Δ*crpA* mutants and measured by lactate dehydrogenase (LDH) activity. The statistical analysis was performed using a two-sided one-way ANOVA (Tukey’s test) for multiple comparisons. Data are presented as values from three biological experiments with three replicates ±standard deviation, ns = nonsignificant. (**l**) ROS accumulation upon exposure of the wild-type and Δ*crpA* mutants to MUMAs in indicated periods. Phorbol-12-myristate-13-acetate (PMA) was used as a positive control for ROS induction. Data are presented as values from two independent biological repetitions and are expressed as average ±standard deviation. The statistical analysis was performed using a two-sided two-way ANOVA (Dunnett’s test) for multiple comparisons. Adjusted (adj.) *P* values. *adj. 0.0211; **adj. 0.0016 and ****P < 0.0001; ns = nonsignificant.

We have previously identified AFUA_7G01060 (AFUB_087640) as exclusively detected on the *A. fumigatus* conidial surface (14). AFUA_7G01060 encodes a cysteine-rich protein named CrpA that has 343 amino acids (27 cysteine residues; Fig. 1a), a putative signal peptide (amino acids 1 to 20), and a putative epidermal growth factor-like domain from amino acids 125 to 156 (Fig. 1a; SM000181; http://smart.embl-heidelberg.de/). AlphaFold structure prediction shows a two-domain protein (Fig. 1b; https://alphafold.ebi.ac.uk/entry/Q4WAE9). We constructed three independent Δ*crpA* mutants and an overexpression *gpdA::crpA* strain with the strong constitutive *gpdA* promoter from the glyceraldehyde-3-P-dehydrogenase gene (which has about 12-fold more *crpA* mRNA than the wild-type strain; Fig. S1 to S4, https://doi.org/10.6084/m9.figshare.29851538.v1). All three independent *crpA* null mutants display the same phenotypes, strongly suggesting that the observed phenotypes are only due to the *crpA* single deletion and not to secondary mutations present in these strains (Table S1, https://doi.org/10.6084/m9.figshare.29851538.v1). We extensively phenotyped the mutants and found no striking differences between Δ*crpA* and *gpdA::crpA* compared to the wild-type strain (Fig. 1c; Table S1, https://doi.org/10.6084/m9.figshare.29851538.v1). However, Δ*crpA* resting and swollen conidia exhibited increased chitin exposure (measured by Calcofluor White, CFW, which binds and blocks chitin biosynthesis) but lower β−1,3-glucan exposure (assessed via Fc-hDectin-1a staining fluorescence) relative to wild-type (Fig. S5, https://doi.org/10.6084/m9.figshare.29851538.v1).

CrpA has a narrow distribution among several fungal classes, including most of the homologs in *Aspergillus* spp (Eurotiales, class Eurotiomycetes) and very few representatives in the Sordariales, Hypocreales, Xylariales (class Sordariomycetes), Botryospheriales (Dothideomycetes), and Mortierellales (a monotypic fungal order, within the phylum of Zygomycota) (Fig. 1d).

### CrpA is important for cytokine production

To begin to understand the function of the *A. fumigatus* CrpA in fungal pathogenesis, we used *in vitro* models of infection to assess the role of CrpA using both Δ*crpA* and *gpdA::crpA* strains. The *crpA* null mutants, but not *gpdA::crpA,* showed increased killing by murine macrophages (MUMAs) compared to the wild-type strain, but surprisingly, were less phagocytosed by MUMAs than the wild-type (Fig. 1e and f). This could be explained by the fact that the Δ*crpA* mutants, although not statistically significant, have an increased percentage of adherent conidia on the surface of MUMAs that are not phagocytosed (Fig. 1g). When viability (CFUs) is normalized by the phagocytic index, the null and overexpression mutants showed comparable levels of survival to the wild-type strain (Fig. 1h). Survival is decreased in the *crpA* null, but not in the *gpdA::crpA* mutants, when exposed to A549 epithelial cells (Fig. 1i), and there are no differences in A549 epithelial cell adhesion between the *crpA* mutants and the wild-type strain (Fig. 1j). The *crpA* mutants had no significant impact on MUMAs' lactate dehydrogenase (LDH) activity and MUMA survival and also no significant differences in the production of reactive oxygen species (ROS) when compared to the wild-type strain (Fig. 1k and l).

**Fig 2:**
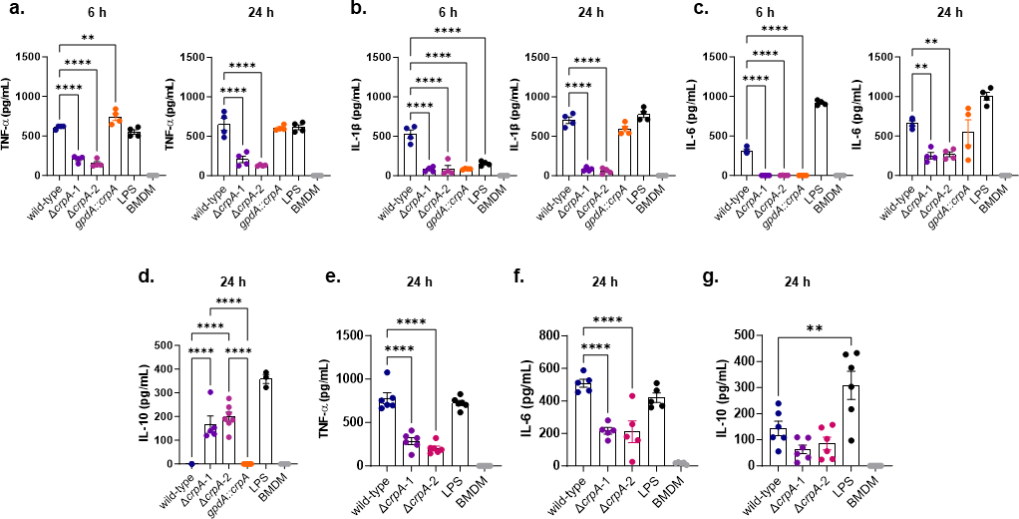
The *A. fumigatus* Δ*crpA* mutant has reduced production of pro-inflammatory cytokines. The Δ*crpA* mutants have reduced TNF-α, IL-1β, and IL-6 and increased IL-10 cytokine production when compared to the wild-type strain. MUMA cells (**a–d**) or alveolar macrophages (**e–g**) were challenged with *A. fumigatus* conidia at a multiplicity of infection (MOI) of 1:10, and after 6 or 24  h of incubation, the cytokine production was measured in the supernatant. Data are presented as values from two independent biological repetitions and are expressed as average ±standard deviation. The statistical analysis was performed using a two-sided two-way ANOVA (Dunnett’s test) for multiple comparisons. Adjusted (adj.) *P* values. *adj. 0.0211; **adj. 0.0016 and ****P < 0.0001; ns = nonsignificant. The statistical analysis was performed using a two-sided one-way ANOVA (Tukey’s test) for multiple comparisons. Data are presented as values from three biological experiments with three replicates ±standard deviation, ns = nonsignificant. Bacterial lipopolysaccharide (LPS) was used as a positive control.

We hypothesized that deletion of CrpA might affect activation and, therefore, cytokine production of infected macrophages. We therefore measured the production of proinflammatory cytokines TNF-α, IL-1β, and IL-6 by MUMAs infected with wild-type, Δ*crpA*, or *gpdA::crpA* strains (Fig. 2a through c). We observed a significant reduction of TNF-α, IL-1β, and IL-6 production by MUMAs infected with Δ*crpA* mutants at 6 and 24 h (Fig. 2a through c). After infection with *gpdA::crpA* spores for 6 h, MUMAs produced significantly more TNF-α, but significantly less IL-1β and IL-6, with these cytokine levels returning to wild-type levels at 24 h (Fig. 2a through c). We also measured IL-10, a canonical anti-inflammatory cytokine, and observed increased production in MUMAs infected with Δ*crpA* mutants at 24 h (Fig. 2d). However, IL-10 was undetectable at 6 h in both wild-type and mutant strains (data not shown). Alveolar macrophages showed that Δ*crpA* mutant infection has reduced TNF-α and IL-6 production, but there are no differences in the IL-10 production (Fig. 2e through g).

### Prostaglandin profiling of MUMAs exposed to Δ*crpA*

**Fig 3:**
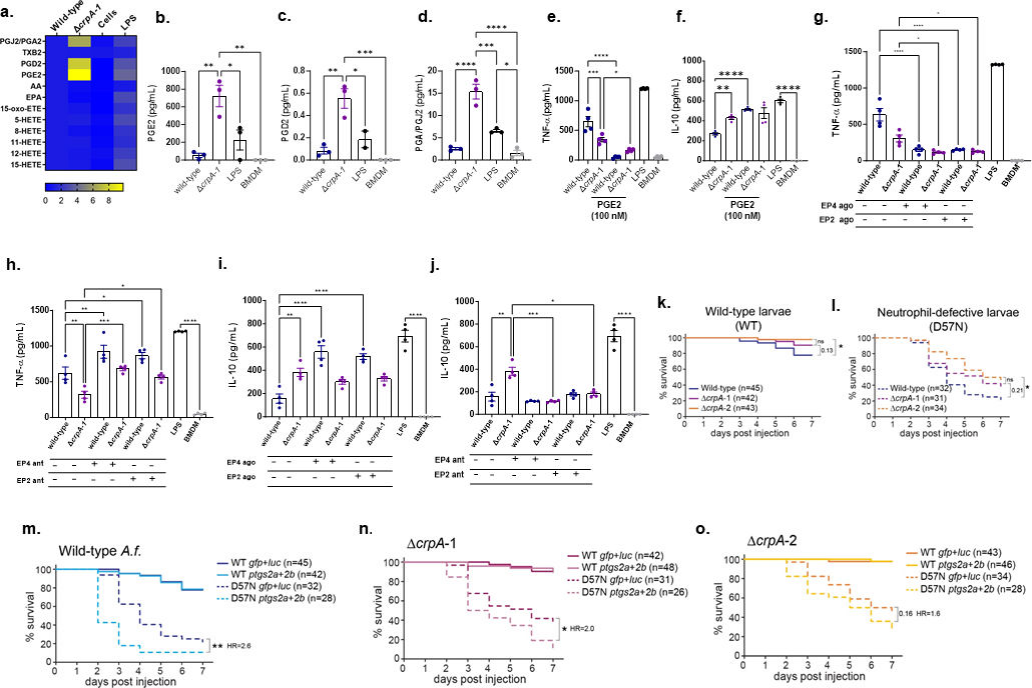
Prostaglandins PGE2, PGD2, and PGA2/PGJ2 have increased production when MUMAs are exposed to the *A. fumigatus* Δ*crpA* mutant strain. (**a**) Heat map of 12 eicosanoids/prostaglandin levels in the wild-type and Δ*crpA-1*. (**b–d**) PGE2, PGD2, and PGA2/PGJ2 production in the wild-type and Δ*crpA-1* mutant strain. (**e and f**) MUMAs were exposed to the *A. fumigatus* wild-type, Δ*crpA*-1, for 24 h in the absence or presence of PGE2 (100 nM), and TNF-α and IL-10 production were measured. (**g–j**) MUMAs were exposed to the *A. fumigatus* wild-type and Δ*crpA-1* mutant for 24 h in the absence or presence of 100 ng/mL of different EP2 and EP4 agonists (TCS2510 and Butaprost) or antagonists (ONO AE3 and PF04418948), and TNF-α and IL-10 cytokine production was measured. The signals (+) and (–) denote the presence or absence of an agonist (Ago) or antagonist (Ant). The results graphic shows the value of three to four technical replicates ±standard deviation. Statistical analysis was performed using a two-sided one-way ANOVA (Dunnett’s test) for multiple comparisons. *P  <  0.05, **P  <  0.01, ***P  <  0.001, and ****P  <  0.0001. Bacterial lipopolysaccharide (LPS) was used as a positive control. (**k–o**) Wild-type or neutrophil-defective (D57N) zebrafish larvae were injected with wild-type or Δ*crpA* spores, and survival was monitored for 7 days. Total N for each condition across three independent replicates is noted. Average infection doses from CFU plating: WT *gfp +luc* + WT = 61; WT *ptgs2* +WT = 48; D57N *gfp +luc* + WT = 45; D57N *ptgs2* +WT = 66; WT *gfp +luc* + Δ1 = 43; WT *ptgs2* + Δ1 = 24; D57N *gfp +luc* + Δ1 = 56; D57N *ptgs2* + Δ1 = 43; WT *gfp +luc* + Δ2 = 51; WT *ptgs2* + Δ2 = 42; D57N *gfp +luc* + Δ2 = 32; D57N *ptgs2* + Δ2 = 37.

Eicosanoids, lipid metabolites of arachidonic acid (AA), including prostaglandins (PGs) and leukotrienes (LTs), have emerged as potent endogenous mediators and modulators of innate immunity, including through modulation of cytokine production (16–18). Since we found that macrophages exposed to Δ*crpA* mutants have decreased proinflammatory cytokine production, we hypothesized that Δ*crpA* spores might also alter macrophage production of eicosanoids. We exposed MUMAs to wild-type and Δ*crpA-1* mutant spores for 24 h and performed target-mass spectrometry identification for 12 eicosanoids extracted from the MUMA supernatants. We observed an increased production of eicosapentaenoic acid (EPA), PGD2, PGE2, and 15-oxo-5,8,11,13-(Z,Z,Z,E)-eicosatetraenoic acid (15-oxo-ETE) when MUMAs were exposed to *A. fumigatus* wild-type (Fig. 6, https://doi.org/10.6084/m9.figshare.29851538.v1). After stimulation with the Δ*crpA-1* strain, MUMAs produced significantly more PGE2, PGD2, and the corresponding metabolized products PGA2/PGJ2 compared to stimulation with wild-type spores, with increases of about 8-, 5-, and 5-fold, respectively (Fig. 3a through d).

### Δ*crpA*-modulated PGE2 drives changes in cytokine production

As PGE2 is the most well-characterized of these prostaglandins and is known to directly affect cytokine production, we next tested whether treatment with PGE2 affects cytokine production by infected MUMAs. MUMAs were infected either with *A. fumigatus* wild-type or Δ*crpA-1* mutant and were exposed or not to PGE2 (100 nM), and cytokine production was measured (Fig. 3e and f). As previously shown, TNF-α and IL-10 production are lower and higher, respectively, from MUMAs infected with the Δ*crpA-1* mutant compared to the wild-type strain (Fig. 3e and f). In the presence of PGE2, MUMAs infected with both wild-type and Δ*crpA-1* strains have decreased TNF-α production (Fig. 3e). IL-10 production is increased in the wild-type strain when exposed to PGE2, but there are comparable IL-10 levels in the Δ*crpA-1* mutant exposed or not to PGE2, probably because of the high levels of PGE2 already produced by the mutant (Fig. 3f). These data suggest that moderate levels of PGE2 produced by infection with wild-type spores are proinflammatory, leading to TNF-α production and suppression of IL-10. On the other hand, high levels of PGE2, either through infection with a Δ*crpA* strain or by exogenous PGE2 addition, are anti-inflammatory, leading to decreased TNF-α production and increased IL-10 production.

If modulation of macrophage PGE2 by Δ*crpA* modulates macrophage cytokine production, then we would hypothesize that inhibiting macrophage PGE2 production or signaling would also affect cytokine production. Indomethacin is a nonspecific and reversible inhibitor of both forms of the cyclo-oxygenase (COX) enzyme or prostaglandin G/H synthase, which produces PGE2, but with greater selectivity for COX-1, binding to the enzyme’s active site and preventing the interaction between the enzyme and its substrate (https://go.drugbank.com/drugs/DB00328). We exposed MUMAs to wild-type, Δ*crpA-1*, Δ*crpA-2*, and *gpdA::crpA* strains and then treated them with indomethacin ( Fig. S7a through c, https://doi.org/10.6084/m9.figshare.29851538.v1). In all infected conditions treated with indomethacin, TNF-α levels are low, and IL-10 levels are high, but no effects were observed on IL-6 production (Fig. S7a through c, https://doi.org/10.6084/m9.figshare.29851538.v1). These data support the conclusion that abolishment of all PGE2 production leads to lower pro-inflammatory TNF-α production and higher anti-inflammatory IL-10 production.

The EP2 and EP4 prostanoid receptors belong to the family of G-protein-coupled receptors and are two of the four subtypes of receptors for prostaglandin E2 (PGE2) (19–24). They couple to G-proteins and increase intracellular cAMP formation (19–24). To determine if PGE2 signaling through EP2 and/or EP4 modulates cytokine production by MUMAs infected with wild-type or Δ*crpA* spores, MUMAs were exposed to 100 nM of agonists or antagonists of EP2 [agonist EP2, butaprost (methyl 7-[(1R,2R,3R)−3-hydroxy-2-[(E,4R)−4-hydroxy-4-(1-propylcyclobutyl)but-1-enyl]−5-oxocyclopentyl]heptanoate) and antagonist EP2, PF-04418948 [1-(4-fluorobenzoyl)−3-[(6-methoxynaphthalen-2-yl)oxymethyl]azetidine-3-carboxylic acid] or EP4 [agonist EP4 TCS2510 (5R)−5[(3S)−3-hydroxy-4-phenyl-1-buten-1-yl]−1-[6-(2H-tetrazol-5-yl]−2-pyrrolidinone and antagonist EP4, ONO-AE3-208 [4-[4-cyano-2-[2-(4-fluoronaphthalen-1-yl)propanoylamino]phenyl]butanoic acid] (25) in the presence of *A. fumigatus* wild-type or Δ*crpA-1* mutant, and cytokine production was measured (Fig. 3g through j). TNF-α production has lower and higher levels when MUMAs infected with wild-type and Δ*crpA-1* mutants are exposed to EP2 or EP4 agonists and antagonists, respectively, compared to untreated MUMAs infected with wild-type (Fig. 3g and h). In contrast, IL-10 production is higher from MUMAs infected with the wild-type strain after exposure to EP2 and EP4 agonists. MUMAs infected with Δ*crpA* have the same IL-10 levels with and without treatment with EP2 and EP4 agonists (Fig. 3i), likely because these cells already produce high levels of PGE2 due to Δ*crpA* infection, similar to results when external PGE2 was supplied (Fig. 3e and f). When MUMAs infected with the Δ*crpA* mutant are exposed to EP2 and EP4 antagonists, IL-10 production is lower, supporting the conclusion that high levels of PGE2 produced in this infection drive IL-10 production (Fig. 3j). However, EP2 and EP4 antagonists have no significant effect on IL-10 production by MUMAs infected with the wild-type strain since in this situation, IL-10 production is already low (Fig. 3j).

These results suggest that *A. fumigatus* infection in MUMAs induces PG production of EPA, PGD2, PGE2, and 15-oxo-ETE, and there is a direct correlation between the production of PGE2 and modulation of cytokines. The Δ*crpA* mutant has higher production of these PGs than the wild-type, and our results suggest that the decreased and increased production of pro-inflammatory cytokines and IL-10, respectively, could be due to PGE2 production. Moreover, this process is activated through the EP2 and EP4 receptors.

To determine the impact of Δ*crpA* prostaglandin modulation on *A. fumigatus* pathogenesis in an animal model, we used the larval zebrafish infection model in which cyclooxygenase signaling and PGE2 production have recently been shown to promote control of infection (26). In both wild-type and neutrophil-defective larvae, Δ*crpA* mutants are less virulent than a wild-type strain, although this difference is only statistically significant for Δ*crpA*-2 (Fig. 3k and l). To assess the role of prostaglandin production in this virulence difference, we used CRISPR/Cas9 to target *ptgs2a* and *ptgs2b*, genes encoding the enzyme cyclooxygenase-2 (COX-2), which is required for infection-induced PGE2 production. In neutrophil-defective larvae, COX-2 deficiency makes larvae 2.6 times more likely to succumb to wild-type infection, a significant difference (Fig. 3m). In wild-type larvae, PGE2 is induced in response to infection (20), but this is balanced by the inhibition of this pathway by CrpA, and overall, PGE2 levels are protective. However, COX-2 deficiency only increases the susceptibility to Δ*crpA* infection by 2.0- and 1.6-fold, suggesting that when host PGE2 cannot be induced, CrpA is less important for virulence (Fig. 3n and o). In fact, in neutrophil-defective larvae infected with Δ*crpA*-2, COX-2 deficiency does not significantly affect larval susceptibility to infection (Fig. 3o). We next directly compared situations in which we predict there to be similar PGE2 levels: control larvae infected with wild-type spores in which PGE2 levels are controlled by CrpA, and COX-2-deficient larvae infected with Δ*crpA* spores in which PGE2 levels will also be low. In neutrophil-defective larvae, these comparisons with both Δ*crpA*-1 and −2 are not statistically significant. Overall, these results suggest that modulation of PGE2 production and signaling is a major pathway through which CrpA deficiency modulates host immune function.

### High-resolution magic-angle spinning solid-state nuclear magnetic resonance (MAS NMR) spectroscopy to characterize the cell wall of the Δ*crpA* swollen conidia

**Fig 4:**
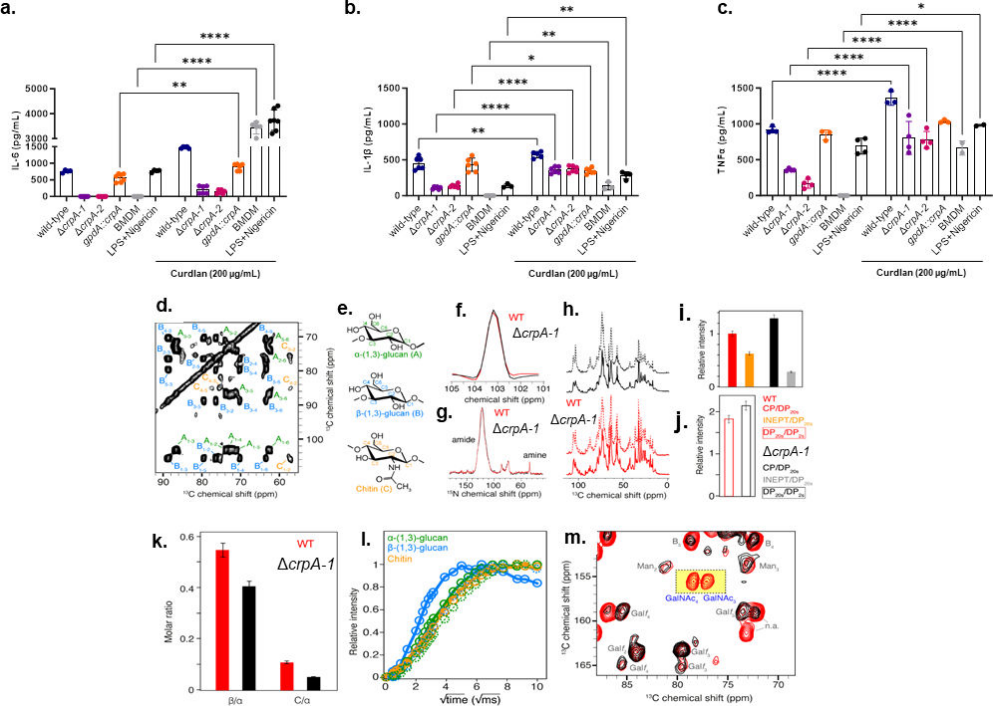
Solid-state NMR characterization of the Δ*crpA* cell wall in swollen conidia. (**a–c**) The Δ*crpA* mutants have reduced TNF-α, IL-1β, and IL-6 and increased IL-10 cytokine production when compared to the wild-type strain. MUMA cells were challenged with *A. fumigatus* conidia at a multiplicity of infection (MOI) of 1:10 in the absence or presence of curdlan (200 µg/mL), and after 24  h of incubation, the cytokine production was measured in the supernatant. Data are presented as values from two independent biological repetitions and are expressed as average ±standard deviation. The statistical analysis was performed using a two-sided two-way ANOVA (Dunnett’s test) for multiple comparisons. Adjusted (adj.) *P* values. *adj. 0.0211; **adj. 0.0016 and ****P < 0.0001; ns = nonsignificant. Bacterial lipopolysaccharide (LPS) was used as a positive control. (**d**) 2D ^13^C-^13^C correlation experiment recorded at 500 MHz and 11 kHz MAS, used to identify the main rigid polysaccharides of *A. fumigatus* cell wall. (**e**) Chemical structure of a-, b-1,3-glucan and chitin. (**f**) Comparison of ^13^C line width for a-1,3-glucan (C_1_ signal). (**g**) ^15^N cross-polarization experiments. (**h**) Comparison of direct ^13^C polarization experiments recorded with a recycle delay of 2 sec (plain line) and 20 sec (dashed line). (**i**) CP/DP intensity ratio measured for WT (red) and Δ*crpA* (black). INEPT/DP intensity ratio measured for WT (orange) and Δ*crpA* (gray). (**j**) Intensity ratio between direct ^13^C polarization recorded with 20 and 2 sec recycle delay, for WT (red) and Δ*crpA* (black). Cell wall polysaccharide reorganization in Δ*crpA* swollen conidia. (**k**) Polysaccharides' molar ratio as measured by NMR signal intensity, for α−1,3-glucan/β−1,3-glucan (b/a) and chitin/α−1,3-glucan (C/a). WT in red, Δ*crpA* in black. Error bar calculation is provided in the Materials and Methods section. (**l**) Polysaccharides' water accessibilities as measured by build-up magnetization curves. Plain line (WT) and dashed line (Δ*crpA*). (**m**) Mobile polysaccharides of the cell probed by 2D ^13^C-^13^C DP-INADEQUATE (WT: red, Δ*crpA*: black). Signals of N-acetyl-galactosamine (GalNAc) are highlighted in yellow.

One mechanism through which Δ*crpA* could drive increased PGE2 production and altered cytokine production by macrophages is through a difference in pathogen-associated molecular patterns (PAMP) recognition and pattern recognition receptors (PRR) signaling. We therefore wondered if alterations in cytokine production could be rescued by exogenous PAMP stimulation. The addition of β−1,3-glucan (curdlan 200 µg/mL) significantly increased IL-6, IL-1β, and TNF-α production by MUMAs infected with wild-type or Δ*crpA* spores, demonstrating that exogenous dectin-1 receptor stimulation can partially rescue cytokine production by Δ*crpA*-infected macrophages (Fig. 4a through c).

Our results suggest that Δ*crpA* conidia fail to fully activate certain MUMA PRRs (e.g., dectin-1, which recognizes β−1,3-glucan), potentially contributing to the observed reduction in proinflammatory cytokine production. This aligns with our phenotypic analysis showing that Δ*crpA* conidia exhibit increased chitin exposure but decreased β−1,3-glucan levels (Fig. S5, https://doi.org/10.6084/m9.figshare.29851538.v1), further supporting a role for CrpA in modulating cell wall organization. As a preliminary step to understand if CrpA can impact the cell wall composition and organization, we investigated the wild-type and Δ*crpA* cell walls by using *in situ* magic angle spinning nuclear magnetic resonance (MAS NMR) on intact conidia following the strategy presented here (27). We identified the three main polysaccharides of the cell wall in both strains (Fig. 4d and e). The ^13^C line widths are comparable, suggesting that the structural order of polysaccharides is conserved between the wild-type and the Δ*crpA* mutant (Fig. 4f). The intensity ratio between amine (~32 ppm) and amide (~120 ppm) signals is also conserved, indicating that the ratio of chitosan/chitin, i.e*.,* the degree of deacetylation, is comparable (Fig. 4g). The comparison of various NMR polarization transfers (Fig. 4h), namely, direct ^13^C polarization (DP) with a long recycle delay (20 sec), cross-polarization (CP), and J-coupling transfer (INEPT), suggests a global reshuffling of the cell wall, associated with an increase in rigid cell wall polysaccharides, and a decrease in mobile polysaccharides (i.e., galactomannan and galactosaminogalactan) for the Δ*crpA* mutant (Fig. 4h through j). The molar ratio between the three rigid polysaccharides indicates a clear reshuffling, with less β−1,3-glucan and chitin in the Δ*crpA* mutant (Fig. 4k), corroborating what was shown before (in Fig. 4i and j), where we observed that the proportion of rigid/mobile is changed. We determined the polysaccharides' water accessibilities, as measured by build-up magnetization curves (Fig. 4l). The major change is observed for β−1,3-glucan, exhibiting a higher water accessibility for the WT since the build-up for the Δ*crpA* mutant decreases to reach the same trend observed for α-glucan and chitin, suggesting that in the mutant, β-glucan is less exposed to the surface compared to the wild-type. We also compared the presence of polysaccharides embedded in the mobile cell wall component (i.e*.,* mannose, galactofuranose, Galf, and galactosamine, GalN, and N-acetyl-D-galactosamine, GalNAc), and we observed a main difference at the level of GalNAc, which is not observed for the mutant (Fig. 4m), suggesting that surface polysaccharides (mainly GAG) can also be impacted in the Δ*crpA* mutant.

Collectively, these results suggest that Δ*crpA* mutants have an altered polysaccharide composition of the cell wall and induce cell wall reshuffling, which can impact the conidial recognition by MUMAs.

### CrpA:GFP is secreted into macrophages

**Fig 5:**
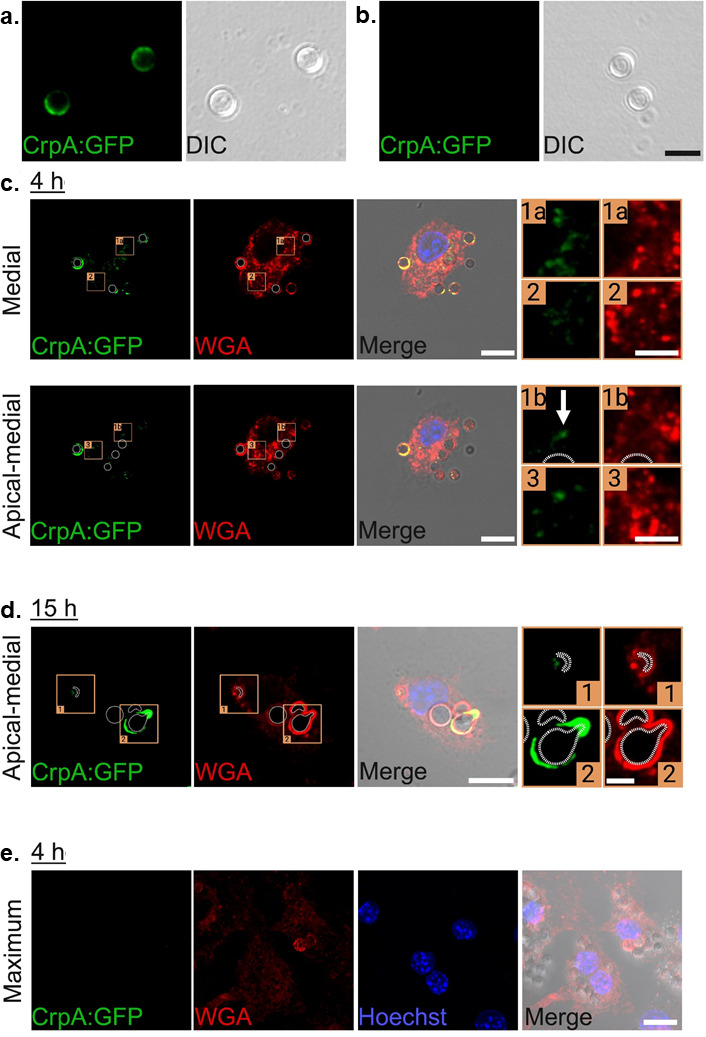
CrpA:GFP is localized on *A. fumigatus* swollen conidia and intracellularly in infected MUMAs. CrpA:GFP *A. fumigatus* conidia were seeded onto BioBond-coated glass slides for 4 h, fixed, and incubated with the primary rabbit IgG anti-GFP-Alexa Fluor-594 antibody, followed by the fluorescently labeled secondary antibody (green). (**a**) Confocal microscopy revealed that CrpA:GFP was evenly distributed on the swollen conidia, with moderately concentrated labeling in specific regions. A weaker labeling can also be observed in the cytoplasm. (**b**) No fluorescence signal was detected in control samples incubated only with the secondary antibody. Goat IgG anti-Rabbit IgG conjugated to Alexa Fluor-594 was used as the secondary antibody in all samples. Scale bar: 5 nm. MUMAs were infected with *A. fumigatus* conidia expressing CrpA:GFP for the indicated time points. Fixed samples were immunolabeled with rabbit anti-GFP-Alexa Fluor-594, followed by anti-rabbit IgG-Alexa Fluor 594 (green). Fungal wall and host cell membranes were stained with wheat germ agglutinin (WGA) conjugated to tetramethylrhodamine (TRITC; red), and nuclei were counterstained with Hoechst 33342 (blue). (**c**) At 4 h post-infection, CrpA:GFP was enriched around nearly all conidia, partially overlapping with WGA in a ring-like pattern. A punctate CrpA:GFP signal was also detected throughout the MUMA cytoplasm near phagocytosed fungi. Insets (1 a, 2, and 3) highlight granular CrpA:GFP structures; Inset 1b shows a filamentous extension (white arrow) emerging from a conidium with the concentrated CrpA:GFP signal. (**d**) At 15 h, CrpA:GFP signal diminished in most conidia, and cytoplasmic granules became scarce. Inset 1 shows loss of signal in a representative conidium, while inset 2 reveals CrpA accumulation at the poles of a swollen conidium with an emerging protrusion. (**e**) No signal was observed in control MUMAs infected for 4 h and processed only with secondary antibody. Images represent maximum-intensity Z-projection of 2 slices (A – medial: slices 8–9; A – apical-medial: slices 12–13). Dotted lines mark internal borders of internalized conidia. Merged panels include all channels and DIC. The inset shows the boxed regions at higher zoom. Scale bar: 10 nm. Scale bar in inset: 3 nm.

We previously constructed a functional CrpA:GFP strain that showed a low GFP fluorescence near the surface of resting and swollen conidia, but no detectable signal present in germlings (14). We demonstrated that CrpA:GFP is a secreted protein, suggesting the signal peptide observed in this protein is functional (14). Considering CrpA is a secreted protein, we investigated the CrpA:GFP localization when conidia infected MUMAs by using immunofluorescence. First, to investigate the presence and distribution of CrpA on the *A. fumigatus* swollen conidia surface, fixed CrpA:GFP conidia were seeded onto pretreated glass slides. After 4 h of incubation, CrpA exhibited a broad distribution pattern on the fungal cell surface (Fig. 5a). The medial section of confocal imaging showed moderately concentrated labeling in specific regions of the conidia surface and some weaker internal staining, confirming a donut-like labeling for CrpA (Fig. 5a). No signal was detected in control samples incubated with the secondary antibody alone, confirming the specificity of immunostaining (Fig. 5b).

Next, we sought to investigate the subcellular localization of CrpA:GFP during infection. Several distinct localization patterns were observed. In MUMAs infected for 4 h, CrpA:GFP was consistently detected in nearly all conidia and showed a partial overlap with wheat germ agglutinin (WGA) in a characteristic ring-like pattern (Fig. 5c). Interestingly, CrpA:GFP was also distributed in nonfungal structures, displaying a punctate pattern dispersed throughout the MUMA cytoplasm, particularly near the engulfed *A. fumigatus* (Fig. 5c). At higher magnification, CrpA:GFP could be resolved as a distinct granular pattern (Figure A, insets 1 a, 2, and 3). Notably, we observed a bright, filamentous structure extending from the peripheral rim of a conidium (Fig. 5c). After 15 hours of infection, our images suggested that most conidia had lost CrpA:GFP signal, and the granular distribution became rare (Fig. 5c). However, in some engulfed conidia that had begun to swell, we observed the emergence of a protrusion where the CrpA:GFP signal was markedly brighter and appeared to accumulate on opposite sides of the fungal particle (Fig. 5c). As a control, infected cells incubated only with the secondary antibody showed no CrpA:GFP signal (Fig. 5d). These results strongly indicate CrpA:GFP is secreted into the MUMAs.

### CrpA protein directly modulates macrophage PGE2 and cytokine production

**Fig 6:**
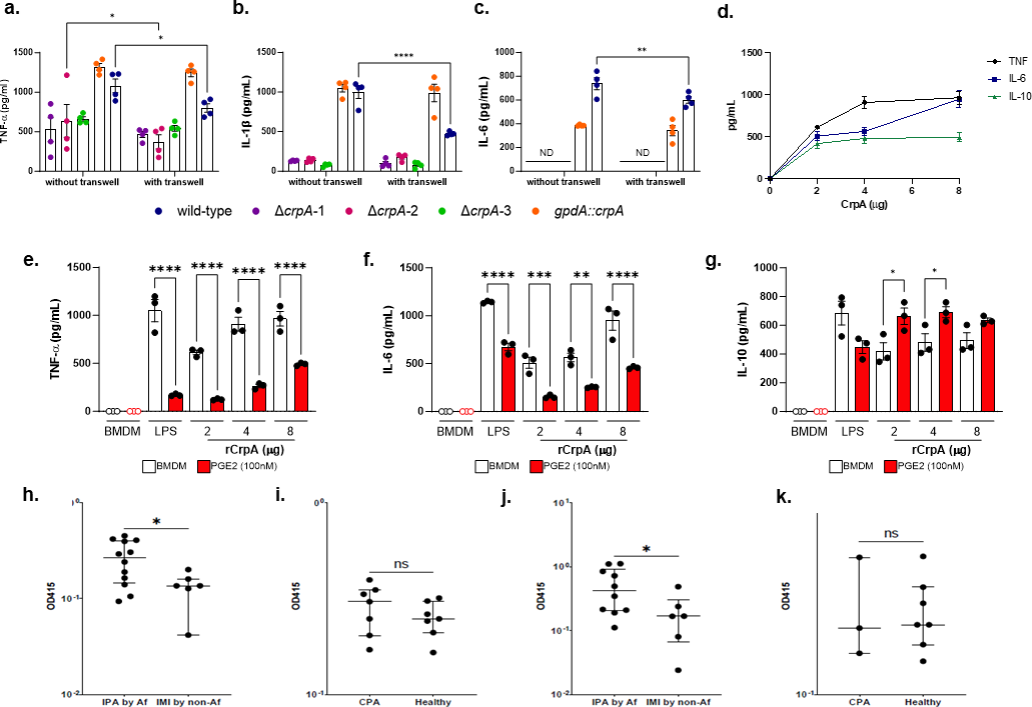
rCrpA can modulate cytokine production. (**a–c**) Transwell migration assays. Wild-type and Δ*crpA* mutants were exposed to MUMAs, and TNF-α, IL-1β, and IL-6 production were measured. The fungus was inoculated into the upper part of the transwell, while the MUMAs were in the lower part. "With transwell" means when the transwell insert was added to the well plate, and "Without transwell" means the well plate without transwell insert. The results are the average of two independent biological repetitions and are expressed as average ±standard deviation. Statistical analysis was performed using a two-sided two-way ANOVA (Šidák’s test) for multiple comparisons, ****P < 0.0001; ns = nonsignificant. (**d**) CrpA can modulate cytokine production. MUMAs were exposed to increasing concentrations of recombinant CrpA (rCrpA) for 24 h at 37°C, and TNF-α, IL-1β, and IL-6 cytokine production was measured. Data are presented as values from two independent biological repetitions and are expressed as mean ± standard deviation. (**e–g**) MUMAs were exposed to increasing rCrpA concentrations in the presence or absence of PGE2, and cytokine production was measured after 24 h. Bacterial lipopolysaccharide (LPS) was used as a positive control. (**h–k**) Humoral IgG response in humans to recombinant *A. fumigatus* CrpA and AspA. CrpA-binding ELISAs were performed with diluted serum samples (1:16) of 12 patients with IPA by *A. fumigatus* (Af), 6 patients with IMI by other molds, 7 patients with CPA, and 7 healthy controls. AspA-binding ELISAs were performed with diluted serum samples (1:256) of 10 patients with IPA by Af, 6 patients with IMI by other molds, 3 patients with CPA, and 7 healthy controls. The median, upper, and lower quartiles are indicated as horizontal lines. Each data point represents the technical mean of two biological replicates from one individual. Significances were tested by the Mann-Whitney test. *P  <  0.05, ns = nonsignificant.

In addition to alterations in the cell wall in Δ*crpA* spores potentially affecting macrophage signaling and activation, CrpA is also a putative secreted protein (14), and we wondered whether this protein could also directly modulate macrophage activation. Transwell migration assays showed that most of the reduced production of TNF-α, IL-1β, and IL-6 by macrophages stimulated with Δ*crpA* mutants does not require contact with MUMAs (Fig. 5a through c). Interestingly, the production of these cytokines is reduced when the wild-type is grown in the absence of contact with MUMAs, suggesting that the sum of the cytokine product is the result of contact combined with effector secretion (Fig. 6a through c).

We heterologously expressed CrpA in *Pichia pastoris* (Fig. S8a, https://doi.org/10.6084/m9.figshare.29851538.v1). Recombinant CrpA (rCrpA) can modulate TNF-α, IL-6, and IL-10 production by using different CrpA concentrations in a dose-response relationship (Fig. 6d). This result is consistent with the conclusion that CrpA stimulates macrophages, leading to lowered proinflammatory cytokine production in macrophages infected with Δ*crpA* spores compared to wild-type spores. Boiling of rCrpA for 10 minutes did not decrease the TNF-α and IL-6 production (Fig. S8b, https://doi.org/10.6084/m9.figshare.29851538.v1). The cytokine induction by rCrpA is not related to its glycosylation since exposure of rCrpA to peptide: N-glycosidase F (PNGase F) did not abolish this induction (Fig. S8b, https://doi.org/10.6084/m9.figshare.29851538.v1). The addition of 8 µg of rCrpA to the Δ*crpA-1* mutant can increase the TNF-α production to levels comparable to the wild-type when exposed to MUMAs (Fig. S8c, https://doi.org/10.6084/m9.figshare.29851538.v1).

 Since we previously found that the altered cytokine production by macrophages infected with Δ*crpA* spores is due to alterations in PGE2 production and signaling, we next wondered whether rCrpA directly impacts PGE2 production and/or activity. To test this hypothesis, we incubated MUMAs with increasing amounts of rCrpA in the absence or presence of PGE2 and measured proinflammatory (TNF-α and IL-6) and anti-inflammatory IL-10 production (Fig. 6e through g). TNF-α and IL-6 production are increased in the presence of LPS but decreased when LPS is incubated with PGE2 (Fig. 6e through g), demonstrating that high concentrations of PGE2 are anti-inflammatory, consistent with previous results (Fig. 3e and f). Similarly, increasing amounts of rCrpA increase the production of TNF-α, IL-6, and IL-10, as previously shown (Fig. 6d through g). However, as with LPS, exogenous PGE2 modulates TNF-α, IL-6, and IL-10 production (Fig. 6e through g). Overall, these data demonstrate that exogenous CrpA can directly modulate macrophage secretion of cytokines, potentially through moderate PGE2 production, but that high levels of PGE2 are anti-inflammatory, leading to lowered TNF-α and IL-6 production and increased IL-10 production.

### Anti-CrpA antibody response in clinical patient samples

If CrpA is a secreted virulence factor in *A. fumigatus*, then we would predict that human patients would generate antibody responses to this antigen. We identified patients who were hospitalized at University Hospital Cologne between 2021 and 2024 and diagnosed with proven or probable invasive mold infection (IMI) or with CPA. To decipher if disease with *A. fumigatus* leads to the development of anti-CrpA antibodies, we measured CrpA antibody levels by serum ELISA in adult patients with IPA by *A. fumigatus* (*n* = 12) and CPA patients (*n* = 7). We used sera of patients with IMI caused by other molds, including other *Aspergillus* spp. (*n* = 6) and healthy individuals (*n* = 7) without any history of IMI or CPA as control groups with similar immune status.

The highest IgG levels were found in antisera from individual patients with IPA by *A. fumigatus*, the second highest in individual CPA patients (Fig. 6h and i), suggesting the role of CrpA in the immune response against *A. fumigatus*. Patients with severe immunosuppression are particularly prone to IMI. To take the effect of immunosuppression on the humoral response into account, we compared the patient cohort with IPA by *A. fumigatus* with a cohort of patients with a similar level of immunosuppression, diagnosed with IMI by other molds. We observed that the median OD415 was significantly higher in the IPA by the *A. fumigatus* cohort (Fig. 6h). The CrpA antibody response was also detected in the serum of healthy individuals, suggesting the role of continuous environmental exposure to *A. fumigatus*. Compared to individuals with CPA, an *A. fumigatus* disease in patients with a relatively intact immune system, these individuals had, on median, higher antibody responses than healthy individuals (Fig. 6i), again suggesting an active host-pathogen interaction involving CrpA during *A. fumigatus* disease. Interestingly, the same pattern was observed for recognition of AspA, a novel glycosyl asparaginase recently identified as a protein involved in fungal evasion and host immunity modulation (14) (Fig. 6j and k).

### CrpA is important for the establishment of virulence in an immunocompetent murine model of aspergillosis

**Fig 7:**
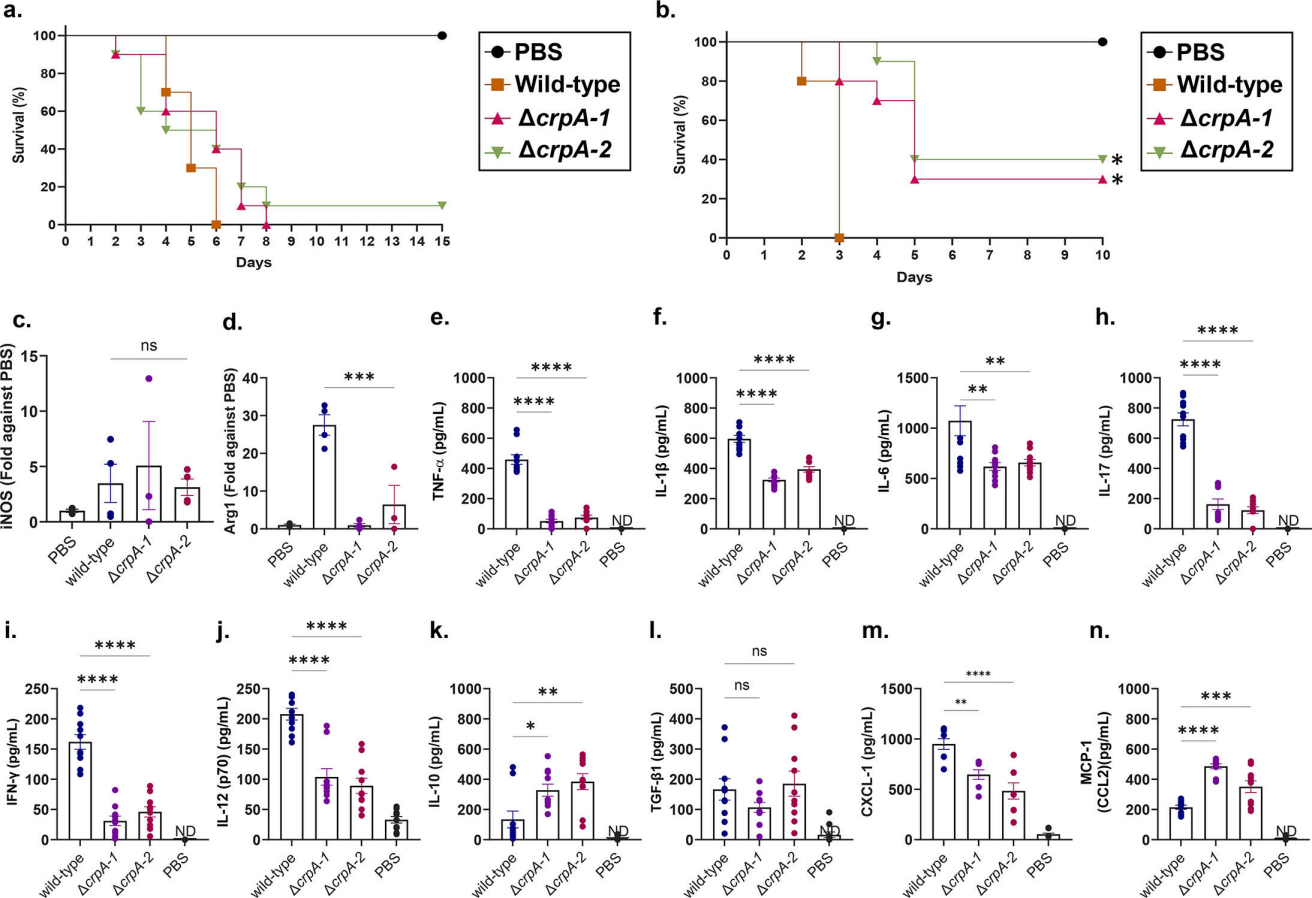
*A. fumigatus* Δ*crpA* mutants are less virulent in the murine immunocompetent infection model. (**a and b**) Survival curve of the wild-type and Δ*crpA* mutants in a murine chemotherapeutic and immunocompetent mouse infection model. The statistical analysis was performed using the log-rank (Mantel-Cox) test. Data are presented as values from *n* = 10 mice per group in two independent experiments, *P < 0.0001 vs WT group. Relative expression of macrophage polarization markers in the pulmonary tissue of immunocompetent mice infected with *A. fumigatus* wild-type or Δ*crpA* mutants. Relative expression of levels of (**c**) iNOS and (**d**) Arg1 mRNAs was determined by quantitative RT- PCR, normalized to β-actin expression. The levels of the relative expression were compared between infected mice and the control group (uninfected/ PBS). Results are expressed as means  ±  SD. Differences were considered significantly different at ***P  <  0.001 and ****P  <  0.0001 relative to uninfected controls and wild type. (**e–n**) Cytokine (TNF-α, IL-1β, IL-6, IL-17, IFN-γ, IL-12, IL-10, and TGF-β1) and chemokine (CXCL-1 and MCP-1) production in the lung homogenates of 3-day infection in an immunocompetent mouse model. The cytokine concentration was normalized per organ mass and is expressed in picograms per milligram of tissue. The injection of PBS alone (uninfected mice) was used for the control group. Data are shown as mean  ±  SD. Statistical analysis was performed using a two-sided one-way ANOVA (Dunnett’s test) for multiple comparisons. *P  <  0.05, **P  <  0.01, ***P  <  0.001, and ****P  <  0.0001 vs the wild-type group.

Overall, our results suggest that deletion of CrpA has a significant impact on the interaction of *A. fumigatus* with host immune cells and pathways. To determine the impact of CrpA deletion on virulence in a mammalian model, we used two different murine models of infection. *A. fumigatus* Δ*crpA* mutants have the same virulence as the wild-type in a chemotherapeutic murine model, but they are less virulent in an immunocompetent murine model, as measured by animal survival (Fig. 7a and b). In the immunocompetent murine model, lung infection with *A. fumigatus* wild-type induces mRNA accumulation of Arg1 and iNOS at levels that are increased and comparable, respectively, to those observed in the PBS control. This pattern suggests a shift toward an Alternatively Activated Macrophage (AAM or M2) response, rather than a Classically Activated Macrophage (CAM or M1) response (Fig. 7c and d). However, the Δ*crpA* mutants have a comparable Arg1 and iNOS mRNA accumulation with the PBS control for both genes (Fig. 7c and d). We measured the production of cytokines TNF-α, IL-1β, IL-6, IL-17, IFN-γ, IL-12, IL-10, and TGF-β1 and chemokines CXCL-1 and monocyte chemoattractant protein 1 (MCP-1/CCL2) in the lung homogenates of the immunocompetent mice infected with the wild-type and Δ*crpA* mutants (Fig. 7e through n). The lack of CrpA caused decreased production of pro-inflammatory cytokines, increased production of the anti-inflammatory cytokine IL-10, and increased production of CCL2 and CXCL-1, which acts as a chemoattractant for several immune cells and is induced upon *A. fumigatus* lung infection (3) (Fig. 7e through o).

Our results indicate that CrpA is important for attenuating the inflammatory responses and chemokine production upon *A. fumigatus* infection in the immunocompetent lungs and plays a role in the modulation of the anti-inflammatory cytokine IL-10.

## DISCUSSION

*A. fumigatus* conidia are the most important infection propagules. Previously, by using a phylogenomic approach, we identified proteins specifically enriched on the surface of *A. fumigatus* conidia, among them a cysteine-rich protein (14). This protein has a signal peptide, and when a functional CrpA:GFP fusion is constructed, this protein is released into the medium and MUMAs, suggesting that CrpA is a secreted protein (14). The precise intracellular location of CrpA into macrophages remains to be determined. We have constructed a series of *crpA* mutants, including null mutants and an overexpression strain. All the phenotypes observed in the overexpression strain are comparable to the corresponding wild-type strain, suggesting CrpA is either not overexpressed at the protein levels or there is a titration effect where CrpA needs other proteins at the same levels to perform its biological functions. This remains to be investigated. When exposed to MUMAs, *crpA* null mutants elicit a decreased production of proinflammatory cytokines and increased production of the anti-inflammatory cytokine IL-10. The Δ*crpA* mutants are less virulent in a murine immunocompetent model, and the same reduced levels of proinflammatory cytokine and increased IL-10 production are observed in the lungs. While *ΔcrpA* induces lower proinflammatory cytokines (TNF-α, IL-1β, and IL-6), it also triggers higher anti-inflammatory IL-10 and PGE2, which suppress overall immune activation. In immunocompetent mice, this balanced response may prevent excessive inflammation-induced tissue damage while still allowing fungal clearance. The *ΔcrpA*’s attenuated virulence likely stems from reduced proinflammatory cytokines (limiting tissue damage) and elevated IL-10/PGE2 (preventing immune overactivation). In contrast, wild-type *A. fumigatus* promotes a dysregulated proinflammatory response that facilitates tissue invasion. This aligns with clinical observations where hyperinflammation worsens IPA outcomes (for reviews, see 1–3).

We applied solid-state NMR to identify possible modifications in the organization of the Δ*crpA* cell wall. This technique is non-destructive and provides high-resolution information that enables the examination of intact cells without the need for chemical perturbation or extraction (for reviews, see (28–30). Recently, this technique has been used to understand the cell wall architecture in *A. fumigatus* mycelia and conidia (27, 31, 32). Here, we observed a significant reduction of β−1,3-glucan and chitin in the Δ*crpA* cell walls, and β−1,3-glucan is less exposed on the Δ*crpA* conidia. This polysaccharide reduction and exposure could be a possible explanation to understand the low levels of proinflammatory cytokines when the mutant is exposed to MUMAs or into the murine lungs. Additional evidence for that is the fact that when MUMAs are exposed to a combination of curdlan, a bacterial β−1,3-glucan, together with Δ*crpA* conidia, high levels of proinflammatory cytokines are induced. Mutants of the genes encoding the previously identified *A. fumigatus* conidial surface proteins CweA and ScwA showed differential susceptibility to known cell wall stressors Congo Red and Calcofluor White (6), suggesting a possible modification in their cell wall organization and/or structure. In these two mutants, the conidial surface protein distribution is similar to the corresponding wild type, while in another mutant of a gene encoding a highly abundant conidial surface protein CcpA, other proteins become more accessible on the conidial surface (9). It remains to be determined if the lack of CrpA affects the protein distribution on the conidial surface. These results strongly indicate that the presence of proteins on resting conidia is important for a normal conidial surface structure, affecting immune recognition by the innate immune system of the host.

We have demonstrated that *A. fumigatus* infection of MUMAs induces the production of four different eicosanoids/prostaglandins, EPA, PGD2, PGE2, and 15-oxo-ETE. Interestingly, the absence of *crpA* increases the expression of two PGs, PGD2 and PGE2, both derived from the PGH2 precursor, and the corresponding metabolized products PGA2/PGJ2. PGA2 is produced through the action of specific PGA synthases, while PGJ2 is formed through the non-enzymatic degradation of PGD2. There is a single report describing PG production by *A. fumigatus* (33). Upon incubating the precursor arachidonic acid ethyl ester, *A. fumigatus* can produce several eicosanoids and prostaglandins (PG), including PGE2, 6-keto-PGF(1α) (the stable hydrolysis product of prostacyclin PGI2), and PGF(2α) (33). Additionally, it generates isoprostanes such as 15(S)−8-iso-PGF(2α) and 15(S)−8-iso-PGE2, as well as thromboxane B2 (TxB2, the stable hydrolysis product of TxA2) (33). These eicosanoids are identical to those produced by cyclooxygenases (COX) in humans; however, the biosynthesis of all these eicosanoids could not be inhibited by the human COX inhibitor indomethacin (33). Two lines of evidence strongly suggest that PGs detected in our work are not produced by *A. fumigatus* but by MUMAs: (i) we demonstrated that indomethacin can inhibit the production of proinflammatory cytokines while increasing the production of the anti-inflammatory cytokine IL-10 in both the wild-type and Δ*crpA* mutant strains, and (ii) specific agonists and antagonists for the PGE2 receptors EP2 and EP4 can modulate the production of TNF-α and IL-10.

We validated the modulation of TNF-α and IL-10 cytokines by using externally applied PGE2 and a combination of PGE2, EP2, and EP4 agonists and antagonists, suggesting that PGE2 is important for *A. fumigatus* MUMA infection. One of the earliest pieces of evidence regarding the role of PGs during infections caused by *A. fumigatus* comes from a study on fungal keratitis (34). In this study, the authors utilized mouse oligo microarrays to compare gene profiles in control corneas with those challenged by heat-inactivated *A. fumigatus* spores. They identified prostaglandin D2 synthase (*Psgd*) as a differentially expressed gene. Furthermore, gene expression studies in human dendritic cells infected with *A. fumigatus* showed an increased expression of genes involved in prostaglandin synthesis (35). The production of PGD2, which is followed by the recruitment of eosinophils into the airways via the Chemoattractant Receptor-Homologous Molecule Expressed on Th2 Cells (CRTH2) receptor, plays a significant role in enhancing airway inflammation and contributing to *Aspergillus*-associated asthma exacerbations (36).

An interesting connection between the reduction of proinflammatory cytokines and the production of PGE2 has been observed in studies involving mice deficient in the IL-33 receptor (Il1rl1–/–). These studies demonstrated that these mice had enhanced lung clearance of *A. fumigatus*. IL-33 acted as a negative regulator of several inflammatory cytokines, as levels of IL-1α, IL-1β, IL-6, IL-17A, and IL-22 were significantly elevated in fungal-exposed Il1rl1−/− mice. Furthermore, when IL-33 was administered to wild-type mice, it reduced the production of fungal-induced IL-17A and IL-22, although it did not affect the levels of IL-1α, IL-1β, or IL-6. The regulation of IL-17A and IL-22 by IL-33 did not involve the modulation of IL-23, but rather related to PGE2 levels. Surprisingly, administering IL-33 in conjunction with PGE2 resulted in a significant increase in PGE2 levels in fungal-exposed *Il1rl1^−/^*− mice, while normal mice produced less PGE2 after fungal exposure. This suggests that IL-33-mediated regulation of IL-17A and IL-22 occurred at the level of PGE2. This finding was supported by *in vivo* inhibition of cyclooxygenase 2, which reduced fungal-induced IL-17A and IL-22 levels, as well as IL-1α, IL-1β, and IL-6 production in *Il1rl1^−/^*^−^ mice, ultimately leading to impaired fungal clearance. The Δ*crpA* mutants have reduced levels of IL-17 in the murine lungs, and a possible correlation between CrpA and IL-33 levels remains to be investigated.

Strong evidence indicates that COX enzyme signaling plays a crucial role in infections caused by *A. fumigatus*, as demonstrated in a zebrafish larva infection model (26). Larvae treated with the pan-COX inhibitor indomethacin exhibit a significantly higher mortality rate from the infection compared to control larvae. COX signaling is essential for promoting the phagocyte-mediated inhibition of both germination and invasive hyphal growth (26). Furthermore, the protective signaling mediated by COX requires the EP2 receptor, and administering exogenous PGE2 has been shown to restore immune control over fungal growth that was diminished due to indomethacin treatment (26). In this study, we find that the increase in larval host susceptibility caused by a deficiency in COX-2 is abrogated in infections with Δ*crpA* strains, further supporting the conclusion that wild-type strains modulate COX activity to increase pathogenesis and that this activity is partially dependent on CrpA. Recently, a study revealed that gliotoxin (GT), a secondary metabolite produced by *A. fumigatus*, triggers a strong production of PGs in resting macrophages, especially in M1 monocyte-derived macrophages (M1-MDMs) (37). GT impairs the functions of activated innate immune cells by selectively suppressing the biosynthesis of the chemotactic lipid mediator leukotriene (LT)B4, while also priming the immune system by promoting PG formation in macrophages. Our work emphasizes the importance of PGs in the regulation of proinflammatory and anti-inflammatory cytokines during *A. fumigatus* infection.

Humans are continuously exposed to *A. fumigatus,* breathing hundreds of conidia every day. We have demonstrated that not only CrpA but also the previously characterized conidial surface protein AspA are recognized by antisera of healthy humans but with higher titers in antisera of IPA patients, suggesting that both proteins are detected by the host and are immunogenic. For other fungi, a large proportion of immunogenic proteins are described to be involved in virulence mechanisms (38), highlighting their importance for fungal pathogenesis and host immunity modulation. Our findings demonstrate that CrpA triggers a strong inflammatory host immune response, promoting the production of proinflammatory cytokines. This strong inflammatory response can contribute to the disruption of lung epithelial integrity, to tissue damage, and subsequently to organ dysfunction and mortality, as shown in the immunocompetent mouse model. Antibodies can mediate different effector functions, including neutralization by binding and Fc-mediated effects including opsonization, activation of the complement system, and antibody-dependent cellular cytotoxicity (ADCC). Since CrpA is involved in a proinflammatory immune response in its secreted form, antibodies may be able to neutralize the proinflammatory properties of CrpA, thereby dampening the immune activation. On the other hand, the humoral immune response may add to the proinflammatory reaction by Fc-mediated effector functions, inflicting additional tissue damage by complement activation or ADCC. This renders immune modulation by antibodies against CrpA a target for further investigation.

Our study demonstrates that CrpA orchestrates immune modulation through both secreted and cell wall-associated functions. NMR-based structural analysis reveals that *crpA* deletion disrupts cell wall architecture, directly linking reduced β−1,3-glucan exposure to impaired immune recognition. These findings position CrpA as a multifunctional virulence factor: it acts as (i) a secreted effector influencing prostaglandin production and (ii) a structural modulator of pathogen-associated molecular pattern (PAMP) presentation. While these dual roles explain the robust attenuation of Δ*crpA* in immunocompetent murine models, the subtler virulence defect in zebrafish suggests host-specific immune interactions that warrant further investigation. Additionally, the precise mechanism of CrpA-induced prostaglandin release—including potential host receptor binding or downstream signaling cascades—remains unresolved. Future studies should address the structural characterization of CrpA-host receptor interactions and therapeutic potential of targeting CrpA-mediated PAMP masking or prostaglandin modulation.

## MATERIALS AND METHODS

### Strains, media, and phenotypic assays

*A. fumigatus* strains A1160, A1163, three *crpA* null mutant strains, Δ*crpA-1*, Δ*crpA-2*, and Δ*crpA-3*, an overexpression strain, *gpdA::crpA*, and a *crpA* fusion with fluorescence green protein strain, CrpA:GFP, were grown in solid minimal medium (MM; 1% [wt/vol] glucose, 50 mL of a 20% salt solution, trace elements, 2% [wt/vol] agar, pH 6.5) at 37°C. The trace elements and nitrate salts were prepared as previously described (39). In the phenotypic characterization, plates containing solid MM were centrally inoculated with 5 µL of 10^6^ spores of each strain in the presence or absence of various stressor agents. After 72 h of incubation at 37°C, radial growth was measured. All plates were grown in triplicate, and the average ± standard deviation (SD) of the data was plotted.

### Construction of *A. fumigatus* mutants

The *crpA* gene was deleted using a gene replacement cassette, which was constructed by *in vivo* recombination in *S. cerevisiae,* as previously described by (40, 41). Thus, approximately 1.0 kb DNA fragments upstream and downstream of the *crpA* coding region were amplified from *A. fumigatus* genomic DNA using the primer pairs pRS Af00700 5'fw/pyrG Af01060 5'rv and pyrG Af01060 3'fw/pRS Af01060 3'rv, respectively. The primers pRS Af00700 5'fw and pRS Af01060 3'rv contained a short homologous sequence to the MCS of the plasmid pRS426. The *pyrG* gene placed within the cassette as a prototrophic marker was amplified from the pCDA21 plasmid using the primers AN4118_5UTR_pyrG_rv and AN4118_3UTR_pyrG_fw. To generate the gpdA:CrpA-3xHA-trpC-pyrG fusion fragment, a 2.6 Kb portion of DNA consisting of the 5-UTR region and CrpA ORF, along with a 1 Kb segment of DNA consisting of the 3-UTR flanking region, was amplified with primers gpdA orf Af01060 fw/HA orf Af01060 rv and pyrG Af01060 3'fw/pRS Af01060 3'rv, respectively, from *A. fumigatus* genomic DNA. The 2.7  kb 3xHA - trpC - pyrG fusion was amplified with primers OZG916/OZG964 from the pOB430 plasmid, and the *gpdA* fragment was amplified with gpdA fw/gpdA rv primers from pFC334 plasmid. For the cassettes CrpA:linker-GFP-trpC-pyrG, the fragments 5-UTR  +  ORF and 3-UTR (1 Kb) were also PCR-amplified from CEA17 genomic DNA with primers pRS Af00700 5'fw/Linker-GFP HA orf Af01060 rv and pyrG Af01060 3'fw/pRS Af01060 3'rv. The linker-GFP-trpC fragment was amplified from the pOB435 plasmid with primers P31/P32, and the *prtA* gene was amplified from the plasmid pPTRI with primers P33/P34. Cassettes were generated by transforming each fragment along with the plasmid pRS426 cut with BamHI/EcoRI into the *S. cerevisiae* strain. The DNA plasmid of the transforming bacteria was extracted, and cassettes were PCR-amplified from these plasmids utilizing TaKaRa Ex Taq DNA Polymerase, which were subsequently transformed into the background of the CEA17, pyrG-. Mutants were selected on MM or MM supplemented with 1  µg/mL pyrithiamine. Southern blot and PCR analysis were performed to confirm all the constructions (Fig. S2 to S4, https://doi.org/10.6084/m9.figshare.29851538.v1). Primers used for the constructions are described in Table S1 (https://doi.org/10.6084/m9.figshare.29851538.v1).

### Production of rCrpA protein

The synthetic gene encoding the recombinant cysteine-rich secreted protein (Afu7g01060) was cloned into the pPICZαA expression vector (Invitrogen). The gene was synthesized by Biomatk Company (CA). The vector contains codons optimized for expression in *Komagataella phaffii*, an N-terminal secretion signal from α-factor, and a Zeocin resistance gene. The resulting plasmid was propagated in *Escherichia coli* DH10β-competent cells (Thermo Fisher Scientific, Waltham, USA), which were selected on low-salt LB agar plates containing 25 µg/mL of Zeocin. The plasmid was purified using the PureYield Plasmid Miniprep System and then linearized with the restriction enzyme Anza 24 MssI (Invitrogen). The linearized plasmid was purified using the Wizard SV Gel and PCR Clean-Up System and used to transform competent *K. phaffii* strain X33 by electroporation (1.5 kV, 25 mF, 200 Ω) in a 0.2 cm cuvette using a MicroPulser Electroporator (Bio-Rad). The recombinant strain was selected, cultured, and induced to express the rCRiSP protein according to the EasySelect Pichia Expression Kit (Invitrogen, Waltham, USA). After 120 hours of induction with 1% (vol/vol) methanol, the culture was centrifuged (8,000 × *g*, 15 min at 4°C), and the supernatant was filtered through a 0.22 µm filter. The filtered material was applied to Con-A Sepharose 4B resin (Cytiva), previously equilibrated in 100 mM Tris-HCl buffer containing 500 mM NaCl (pH 7.4). The sample was eluted with the same buffer containing 500 mM glucose. Protein integrity and sample purity were confirmed by 12% SDS-PAGE. Selected fractions were dialyzed against 1X PBS (pH 7.4) using a Vivaspin 5 kDa centrifugal concentrator (Sartorius), filtered through sterile 0.22 µm membranes, and quantified at 280 nm.

### Murine cell culture

BALB/c bone marrow-derived macrophages (BMDMs) were obtained as previously described (42). Briefly, MUMA cells were cultured for 7–9 days in Dulbecco’s modified Eagle medium (DMEM) 20/30, which consists of DMEM (Gibco, Thermo Fisher Scientific Inc.), supplemented with 20% (vol/vol) fetal bovine serum (FBS) and 30% (vol/vol) L-Cell Conditioned Media (LCCM) as a source of macrophage colony-stimulating factor (M-CSF) on nontreated Petri dishes (Optilux - Costar, Corning Inc. Corning, NY). Twenty-four hours before experiments, BMDMs were detached using cold PBS (Hyclone, GE Healthcare Inc. South Logan, UT). The cell viability and concentration were adjusted using a hemocytometer as previously described (43) diluting 1 part of 0.4% trypan blue (Gibco) and 1 part cell suspension. After counting the unstained (viable) and stained (nonviable) cells separately, the percentage of viable cells was calculated as follows:

Viable cells (%) =total number of viable cells per ml / total number of cells per ml ×100.

For functional assays, 10^6^ cells/mL were added to 24-well tissue culture plates containing the differentiation Eagle Medium (DMEM) 20/30 and allowed to grow adherently overnight at 37°C, 5% (vol/vol) CO_2_. Non-adherent cells were removed by gently washing three times with warm PBS. The viability of the MUMA preparations was >99%, as judged by trypan blue dye exclusion, and the cell percentage is greater than 90% differentiated macrophages.

### Macrophage infection

Bone marrow-derived macrophages (BMDMs) were cultured as described before and were seeded at a density of 10^6^ cells/mL in 24-well plates (Greiner Bio-One, Kremsmünster, Austria). The *A. fumigatus* strains were grown on MM at 37°C for 3–5 days before use. Conidia were harvested with sterile dH2O containing 0.1% (vol/vol) Tween 80 (Sigma-Aldrich), filtered in sterile miracloth (EMD Millipore Corp.), and counted with a hemocytometer. The cells were challenged with the conidia of different strains at a multiplicity of infection of 1:10 and incubated at 37°C with 5% (vol/vol) CO_2_ for different time points. MUMAs were also stimulated with various concentrations of a recombinant CrpA protein (denatured or not) by boiling for 10 min at 100°C and/or Curdlan (Curdlan from Alcaligenes faecalis, Sigma-Aldrich). The lipopolysaccharide (standard LPS, *E. coli* 0111: B4; Sigma-Aldrich, 500 ng/mL) and medium alone were used, respectively, as the positive and negative controls. Cell culture supernatants were collected and stored at −80°C until they were assayed for cytokine or LDH quantification.

### Macrophage internalization and killing assay

BMDMs were seeded at a density of 10^6^ cells/well in 96-well plates and were challenged with conidia of wild-type, *crpA* null mutants, and *gpdA:crpA* strains at a multiplicity of infection of 1:10 and incubated at 37°C with 5% (vol/vol) CO_2_ for 24 h. After incubation, the media was removed, and the cells were washed with ice-cold PBS, and finally, 50 µL of sterile water with 0.1% (vol/vol) Triton X-100 was added to the wells. This suspension was immediately diluted 1:200, and 50 µL was plated on Sabouraud dextrose agar before the plates were incubated at 37°C overnight, and the colonies were counted. Then, 50 µL of the inoculum adjusted to 10^3^ spores/mL was also plated on Sabouraud dextrose agar to correct CFU counts. The CFU/mL for each sample was calculated and compared to that of the A1160 wild-type strain.

### Macrophage phagocytosis and adhesion assays

BMDM cells were prepared as described above. Briefly, 5 × 10^5^ cells/well were seeded in a 24-well plate containing circular glass coverslips and incubated in DMEM supplemented with 10% (vol/vol) FBS at 37°C and 5% (vol/vol) CO2 for 24 h. Then, FITC-labeled conidia were added at an MOI of 1:10. The infection experiment was synchronized for 30 min at 4°C. Unbound conidia were removed by washing with pre-warmed DMEM, and phagocytosis was initiated by shifting the co-incubation to 37°C in a humidified CO_2_ incubator. After 2 h, the phagocytosis was stopped by washing with ice-cold PBS. Labeling of extracellular conidia (adhesion) was performed by incubation with PBS, 0.25 mg/mL calcofluor white (Sigma) for 30 min at 4°C. The cells were washed twice with PBS and fixed with 3.7% (v/v) formaldehyde/PBS for 15 min, followed by two washes with PBS. Microscopic photographs were taken on a Zeiss microscope. For statistical reproducibility, two biological replicates and, in each case, two technical replicates were made and analyzed for each strain. The phagocytic and adhesion index was enumerated by counting 100 macrophage cells per coverslip from duplicate wells. The phagocytic index was calculated by the average number of conidia that had been phagocytosed for each macrophage.

### Transwell assay

BMDMs were seeded at a density of 10^6^ cells/ml in 24-well plates, and the assay was performed with the addition in each well of a transwell insert (0.2 µm filter; Corning) containing the medium alone, the *crpA* null mutants, the overexpression or wild-type conidia at a multiplicity of infection of 1:10 and incubated at 37°C with 5% (vol/vol) CO_2_ for 24 h. After incubation, cell culture supernatants were collected and stored at −80°C for cytokine quantification. In the transwell system, the small and soluble compounds can migrate between the upper transwell and lower chamber, whereas spores are prevented from moving between the chambers.

### Cytokine and LDH quantification

ELISA assay was used to quantify the cytokine levels in MUMA cell supernatant. The quantification of cytokines was performed using Mouse DuoSet ELISA kits (R&D Systems, Minneapolis, MN, USA) according to the manufacturer’s instructions. The plate final absorbance was read at 450 nm, and the cytokine concentration analysis [pg/mL] was performed according to the manufacturer’s instructions, considering the values obtained in the standard curve of each evaluated cytokine. LDH determination was performed using CyQUANT lactate dehydrogenase (LDH) Cytotoxicity Assay (Invitrogen), according to the manufacturer’s instructions. The level of LDH was determined by measuring the absorbance at 490 and 680 nm using a microplate reader (Synergy HTX Multi-Mode, BioTek). All assays were performed in triplicate in at least two independent experiments.

### Epithelial cell culture and infection

The A549 pulmonary epithelial cell line (American Type Culture Collection) was cultured in DMEM containing 10% (vol/vol) FBS (Gemini Bio-Products), and 2 mM L-glutamine with penicillin and streptomycin (Irvine Scientific) in 5% (vol/vol) CO2 at 37°C. Before the assay, 105 A549 cells were cultured in 24-well tissue culture plates overnight. The *A. fumigatus* strains were grown on MM at 37°C for 3–5 days before use. Conidia were harvested with sterile dH2O containing 0.1% (vol/vol) Tween 80 (Sigma-Aldrich), filtered in sterile miracloth (EMD Millipore Corp.), and counted with a hemocytometer. The cells were challenged with the conidia of different strains at an MOI of 1:10 and incubated at 37°C with 5% (vol/vol) CO2 for 24 h. The lipopolysaccharide (standard LPS, *E. coli* 0111: B4; Sigma-Aldrich, 500 ng/ml) and medium alone were used, respectively, as the positive and negative controls.

### Epithelial cell adhesion

To check the conidial adhesion to immortalized lung cells, a total of 10^5^ A549 epithelial cells were plated in DMEM in each well of a 6-well plate culture and let to grow to confluence at 37°C under 5% (vol/vol) CO_2_. Then, the wells were overlaid with fresh DMEM containing 100 wild-type, *crpA* null mutants, or the overexpressed conidia per well and incubated for 2 h at 37°C under 5% (vol/vol) CO_2_. After this, the wells were rinsed three times with pre-warmed PBS, overlaid with Sabouraud dextrose agar, and incubated at 37°C for 48 h. Fungal colonies derived from the conidia that adhered to the cells (or to the wells) were counted. Conidial adherence levels were calculated by dividing the number of adherent conidia by the number of conidia added to the well and expressing the resulting value as a percentage. All experiments were performed in triplicate.

### Zebrafish infection analyses

All experimental procedures of zebrafish embryos and larvae were performed, and adult zebrafish were maintained and handled according to protocols approved by the Clemson University Institutional Animal Care and Use Committee (protocols AUP2024-0270, AUP2025-0094, and AUP2025-0095). Zebrafish of the wild-type AB genotype or neutrophil-defective transgenic line (Tg(*mpx:mcherry-2A-rac2D57N*)) (44) were used. To generate larvae deficient in *ptgs2a* and *ptgs2b*, embryos were naturally spawned and two *in vitro* transcribed guide RNAs (gRNAs) targeting each gene (100 ng/µL each) were injected into 1–4 cell embryos with 250 µg/mL Cas9 protein (PNA Bio, CP01), as previously described (26, 45) (Fig. S9, https://doi.org/10.6084/m9.figshare.29851538.v1). Guide RNAs targeting *gfp* and *luciferase* coding sequences were used as injection controls. Larvae were maintained in E3 medium with methylene blue at 28°C, and embryos were manually dechorionated and anesthetized in 0.3 mg/mL buffered tricaine prior to any experimental procedures. For each experimental replicate, genomic DNA was isolated from individual larvae at 2 days post-fertilization (dpf), and PCR was performed to confirm successful DNA targeting (Fig. S9, https://doi.org/10.6084/m9.figshare.29851538.v1). At 2 dpf, prior to infection, Tg(*mpx:mcherry-2A-rac2D57N*) larvae were screened for mCherry expression on a Zeiss SteREO Discovery.V12 microscope. Wild-type (A1163) and Δ*crpA* spores (strains 1 and 2) were prepared and injected into the hindbrain of 2-dpf larvae as previously described (46). The injection dose was monitored by homogenization and CFU plating of a subset of larvae from each group for each replicate, and the average CFUs are noted in the figure legend. Larvae were rinsed in E3 without methylene blue, transferred to 96-well plates, and monitored for survival for 7 days. Data from three independent replicates were pooled and analyzed by Cox proportional hazard regression in R, which considers variability within and between replicates, to calculate *P* values and hazard ratios (HRs).

### Mouse survival curves and fungal burden

Inbred female mice (BALB/c strain; body weight, 20–22 g; age of 8 to 9 weeks) were housed in vented cages containing five animals. Cages are well-ventilated, softly lit, and subjected to a 12:12 light-dark cycle. The relative humidity was kept at 40 to 60%. Mouse rooms and cages were kept at a temperature range of 22°C. Mice were immunosuppressed with cyclophosphamide (150 mg/kg of body weight), which was administered intraperitoneally on days -4,-1, and 2 before and post-infection (infection day is "day 0"). Hydrocortisone acetate (200 mg/kg body weight) was injected subcutaneously on day −3. Mice (10 mice per group, two repetitions) were anesthetized by halothane inhalation and infected by intranasal instillation of 20 µL containing 1.0 × 10^5^ conidia of *A. fumigatus* wild-type or mutant strains. The viability of the administered inoculum was determined by incubating different serial dilutions of the conidia used in both repetitions on MM medium, at 37°C. As a negative control, a group of 10 mice received PBS only. Animals were sacrificed 15 days post-infection or if moribund. In the immunocompetent model, mice were infected by intranasal instillation of 20 µL containing 5.0 × 10^8^ conidia of *A. fumigatus* wild-type or mutant strains. Animals were sacrificed 10 days post-infection or if moribund.

The principles that guide our studies are based on the Declaration of Animal Rights ratified by UNESCO on 27 January 1978 in its 8th and 14th articles. All protocols adopted in this study were approved by the local ethics committee for animal experiments from the University of São Paulo, Campus of Ribeirão Preto (Permit Number: 23.1.547.60.8; Characterization of virulence and immunopathogenicity of *Aspergillus* spp in the murine model). Groups of five animals were housed in individually ventilated cages and were cared for in strict accordance with the principles outlined by the Brazilian College of Animal Experimentation (COBEA) and Guiding Principles for Research Involving Animals and Human Beings, American Physiological Society. All efforts were made to minimize suffering. Animals were clinically monitored at least twice daily and humanely sacrificed if moribund (defined by lethargy, dyspnea, hypothermia, and weight loss). All stressed animals were sacrificed by cervical dislocation.

### RNA isolation, cDNA synthesis, and real-time PCR analysis

For lung RNA isolation, 100 mg of a murine lung was collected and processed with TRIzol (Invitrogen) and stored at −80°C until the extraction. A total RNA was isolated by TRIzol after cellular lysis and treated with RQ1 RNase-free DNase I (Promega). RNA integrity and concentration were assessed using a NanoDrop Lite Spectrophotometer (Thermo Scientific). For RT-qPCR, the RNA was reverse-transcribed to cDNA using the ImProm-II reverse transcription system (Promega) according to the manufacturer’s instructions, and the synthesized cDNA was used for real-time analysis using the SYBR green PCR master mix kit (Applied Biosystems) in the ABI 7500 Fast real-time PCR system (Applied Biosystems, Foster City, CA, USA). SYBR primers are listed in Table S2 (https://doi.org/10.6084/m9.figshare.29851538.v1). The RLP3 gene was used as a normalizer.

### Reagents for HPLC and standard solutions

HPLC-grade solvents acetonitrile (ACN), methanol (MeOH), and isopropanol were purchased from Sigma-Aldrich (St. Louis, MO, USA) or Merck (Kenilworth, NJ, USA). Ultrapure water (H2O) used in all the preparations was obtained from a MILLI-Q system (Millipore, USA). The acetic acid (99%) and ammonium hydroxide were acquired from Sigma-Aldrich (St. Louis, MO, USA). All eicosanoids, fatty acids, and deuterated internal standards (IS) were purchased from Cayman Chemical Co (Ann Arbor, MI, USA), such as 20-hydroxy Leukotriene B4 (20-OH-LTB4), Leukotriene C4 (LTC4), Prostaglandin B2 (PGB2), 15-keto Prostaglandin E2 (15-keto-PGE2), 20-hydroxy Prostaglandin E2 (20-OH-PGE2), Thromboxane B2 (TXB2), Lipoxin A4 (LXA4), Prostaglandin D2 (PGD2), 6-keto Prostaglandin F1α (6-keto-PGF1α), Prostaglandin E2 (PGE2), 6-keto Prostaglandin F1α-d4 (6-keto-PGF1α-d4), Thromboxane B2-d5 (TXB2-d5), Prostaglandin E2-d4 MaxSpec Standard (PGE2-d4), Prostaglandin F2α-d4 (PGEF2-d4), Lipoxin A4-d5 (LXA4-d5), Resolvin D1-d5 (RvD1-d5), Resolvin D2 (RvD2), Prostaglandin F2α (PGF2α), Prostaglandin G2 (PGG2), Leukotriene C4-d5 (LTC4-d5), Prostaglandin D2-d4 (PGD2-d4), Leukotriene B4 (LTB4), Leukotriene D4 (LTBD4), Leukotriene E4 (LTE4), 6-trans Leukotriene B4 (6-trans-LTB4), 11-trans Leukotriene D4 (11-trans-LTD4), Leukotriene D4-d5 (LTD4-d5), 10(S),17(S)-DiHDHA (PDX), Maresin 1 (MaR 1), Prostaglandin H2 (PGH2), Leukotriene E4-d5 (LTE4-d5), Prostaglandin J2 (PGJ2), 15-deoxy-Δ12,14-Prostaglandin J2 (15-deoxy-PGJ2), (±)5-Hydroxyeicosatetraenoic Acid (5-HETE), Arachidonic Acid (AA), 5-OxoETE, 20-Hydroxyeicosatetraenoic Acid (20-HETE), (±)5(6)-DiHETE, (±)12-Hydroxyeicosatetraenoic Acid (12-HETE), Arachidonic Acid-d8 (AA-d8), 15-deoxy-Δ12,14-Prostaglandin J2-d4 (15-deoxi-PGJ2-d4), 5(S)-Hydroxyeicosatetraenoic Acid-d8 (5-HETE-d8), 5-OxoETE-d7, 12(S)-Hydroxyeicosatetraenoic Acid-d8 (12-HETE-d8), (±)8-Hydroxyeicosatetraenoic Acid (8-HETE), (±)11-Hydroxyeicosatetraenoic Acid (11-HETE), 12-OxoETE, 15-OxoETE, (±)11(12)-DiHET (11,12-DiHETrE), (±)14(15)-DiHET (14,15-DiHETrE), Eicosapentaenoic Acid (EPA), (±)5(6)-DiHET (5,6-DiHETrE), (±)15-Hydroxyeicosatetraenoic Acid (15-HETE), and 15(S)-Hydroxyeicosatetraenoic Acid-d8 (15-HETE-d8). The Solid-Phase Extraction (SPE) Cartridge tube (HyperSep C18-500 mg, 3 mL) was acquired by Thermo Scientific-Bellefonte, PA, USA.

Standard solutions for the calibration curve were carried out as previously reported (47). Briefly, stock solutions of lipid mediators and IS were prepared at 10 µg.mL-1 in MeOH and stored at −80°C. A standard working solution was carried out by a mixture of all the eicosanoids and IS at 100 ng.mL-1 in MeOH/H2O (7:3, vol/vol) containing 0.1% NH4OH by volume. The calibration curves were obtained by successive dilution of the standard working solution in MeOH/H2O (7:3, vol/vol) to the following final concentrations: 2.3, 4.6, 9.2, 18.5, 37.0, 74.0, 148.1, 222.2, 333.3, and 500 ng.mL-1.

### Sample eicosanoids extraction

Solid Phase Extraction (SPE) Cartridge tube (HyperSep C18-500 mg, 3 mL) was acquired by Thermo Scientific-Bellefonte, PA, USA. The extraction of samples was carried out based solid-phase extraction (SPE) protocol as described (47).

Briefly, each sample was spiked with 10 µL of the IS solution before being extracted. Afterward, the samples were centrifuged for 10 min at 4°C and 800 × *g*. Subsequently, the supernatant samples were diluted with water to a maximum solvent concentration of 10%. The next step was carried out by using an extraction manifold (Waters-Milford, MA, USA). The cartridge tube was connected in a manifold and then washed with 4 mL of MeOH and equilibrated with 4 mL of H2O. After loading the diluted samples, the cartridge tubes were again flushed with 5 mL of H_2_O acidified with 0.1% acetic acid to remove hydrophilic impurities. The analytes that had been adsorbed on the SPE sorbent were eluted with 1 mL of MeOH acidified with 0.1% acetic acid and stored at −80°C. At the end of extractions, all collected samples were dried in vacuum systems (SpeedVac SPD 1030, Thermo Fisher Scientific - San Jose, CA, USA) at a pressure of 10.0 Torr and 45°C. The dried samples were resuspended in 50 µL MeOH/H2O (7:3, vol/vol) and analyzed by an LC-MS/MS system.

### Target eicosanoid LC-MS/MS analysis

LC-MS/MS analysis was performed using ultra-high-performance liquid chromatographic (UHPLC - Nexera X2; Shimadzu, Kyoto, Honshu, Japan) coupled to a triple quadrupole time-of-flight (TripleTOF 5600+ -Sciex, Foster, CA, USA) mass spectrometer. The UHPLC system consists of two LC 30AD pumps, an autosampler (SIL-30AC), a CTO-30A oven, a CBM-20A controller, and a DGU-20A degassing unit. The TripleTOF 5600 + mass spectrometer was equipped with a turbo-V IonSpray and calibrant delivery system (CDS). Data acquisition was accomplished on a Shimadzu CBM-20A system interfaced with a computer, using the Analyst TF software version 1.7.1 (SCIEX, Redwood, CA, USA).

The chromatographic separation was carried out using an Ascentis Express C18 column (100 × 4.6 mm; 2.7 µm) from Supelco (Milford, St. Louis, MO, USA). The mobile phases used were phase A (0.2% acetic acid in water with acetonitrile-70:30 vol/vol; pH adjusted at 5.8 with ammonium hydroxide) and phase B (isopropanol with acetonitrile-30:70 vol/vol). The column oven was set at 25°C. Separations were made with a gradient as follows: 0 to 2.0 min, 0% B; 2.0 to 5.0 min, 15% B; 5.0 to 8.0 min, 20% B; 8.0 to 11.0 min, 35% B; 11.0 to 15.0 min, 70% B; and 15.0 to 19 min, 100% B. At 19.0 min, the gradient returned to the initial condition of 0% B, and the column was re-equilibrated until 25.0 min. The injection volume was 10 µL, and the flow rate was set at 0.6 mL min-1.

Mass spectra were acquired from a mass-to-charge ratio (*m/z*) of 50 to 700. The electrospray ionization (ESI) source operated in negative mode was utilized for MRM scanning. MS conditions were as follows: nebulizer gas (GS1) at 50 psi, turbo gas (GS2) at 50 psi, curtain gas (CUR) at 25 psi, electrospray voltage (ISVF) at −4,000V, and turbo ion spray source temperature at 550°C. The dwell time was set at 10 ms, and a mass resolution of 35,000 was achieved at *m/z* 400. External calibrations of the calibrated delivery system (CDS) were carried out using an atmospheric-pressure chemical ionization probe (APCI). Automatic mass calibration (<1 ppm) was performed after each of the five sample injections using APCI Negative Calibration Solution (SCIEX, Redwood, CA, USA) injected via direct infusion at a flow rate of 350 µL/min.

### Bioinformatics and statistical analyses

All data sets acquired were analyzed using the software PeakView 2.1 (Sciex, Foster, CA, USA) and MultiQuantTM 3.0.2 (Sciex, Foster, CA, USA). Statistical analysis and graphics data were performed using GraphPad Prism software version 9.0 (Prism, La Jolla, CA), and the group graphics data were expressed as means ± s.e.m., and statistical significance was set at *P* < 0.05. Partial least squares discriminant analysis (PLS-DA) and heatmaps were determined using the MetaboAnalyst 6.0 online software (https://www.metaboanalyst.ca/).

### Preparation of ^13^C,^15^N-labeled conidia

Fungal stocks were streaked onto an agar slant containing 1% glucose as the carbon source and 5 mM NH₄Cl as the nitrogen source, supplemented with a salt solution and trace elements, and incubated at 37°C for 15 days. Conidia were harvested using 0.5% aqueous Tween-80 and sub-cultured on an agar slant containing 1% ¹³C-glucose, 5 mM ¹⁵NH₄Cl, along with salt solution and trace elements. The culture was incubated at 37°C for 15 days. Conidia were collected using 0.5% aqueous Tween-80, followed by filtration through Miracloth to obtain dormant conidia. To generate swollen conidia, dormant conidia were inoculated into liquid minimal medium containing 1% ¹³C-glucose, 5 mM ¹⁵NH₄Cl, and supplemented with salt solution and trace elements. The culture was incubated at 37°C in a shaking incubator set to 250 rpm for 4 h 30 min. The swollen conidia were collected by centrifugation and fixed by incubation with 2.5% formaldehyde in PBS at ambient temperature for 1 hour, followed by overnight incubation at 4°C. The fixed conidial suspension was then centrifuged (5,500 rpm, 30 min), and the supernatant was discarded. The fixation was quenched with 0.1 M NH₄Cl, thrice. Finally, the conidia were washed twice with Milli-Q water and packed into 4 mm MAS NMR rotors.

### Solid-state NMR analysis

All spectra were recorded at 600 MHz ^1^H frequency (Bruker Biospin spectrometer) using a 4 mm triple channel HCN MAS probe, a spinning frequency of 11 kHz, and high-power ^1^H decoupling (90 kHz). The temperature-dependent position of the water proton resonance was used to set the sample temperature at 5°C (48). Cross-polarization transfers were achieved using a contact time of 1 ms. Direct polarization ^13^C experiments were recorded using recycle delays of 2 and 20 seconds. Water-edited experiments were recorded using a T_2_ filter of 1.5 ms and a variable ^1^H-^1^H spin diffusion period. Two-dimensional (2D) ^13^C-^13^C PDSD spectra were recorded using 224 scans and acquisition times of 7 ms (t_1_) and 15 ms (t_2_). Relative intensity ratios are measured using 1D ^13^C-detected experiments with various polarization transfers; the error bar is 5%. Molar ratios of rigid polysaccharides were determined using cross-peak intensities from the 2D PDSD ^13^C-^13^C correlation spectra, using the approach described here (27). The standard error was determined using the standard deviation of integrated peak volume, dividing the sum of the number of cross-peaks for each specific polysaccharide by the fraction of total standard error, considering the total integrated peak volume. All spectra were analyzed using TopSpin 3.6.1 and CcpNMR (49).

### CrpA phylogenetic analysis

To visualize the distribution of the *crpA* gene presence among fungi, the CrpA amino acid sequence was used for BLAST search (50) against the NCBI RefSeq database (51). The sequences found were aligned using MAFFT v7.508 (52) for the inference of a maximum likelihood phylogenetic tree using IQ-TREE v1.7 (53) with the substitution model JTT + I + G4, determined to be the best by the program. The calculated tree was visualized and edited using iTOL v6 (54).

### Study participant details

Work with human samples was approved by the Ethics Committee at the University of Cologne, Germany (local ethics ID: 08-160), and study conduct was consistent with Good Clinical Practice (GCP) guidelines and the Declaration of Helsinki. For this study, patients with mold disease and healthy controls were enrolled. Only patients with probable/proven invasive mold infection (IMI), including invasive pulmonary aspergillosis (IPA) by *A. fumigatus* (Af) according to the EORTC/MSG 2020 criteria or patients with chronic pulmonary aspergillosis (CPA) diagnosed according to ESCMID/ERS/ECMM consensus definitions 2016 were included in this study (55, 56). All participants gave written informed consent before participating in the study.

### Isolation of serum from whole blood

Serum collection tubes (Sarstedt, Germany) were centrifuged at 2,000 × *g* for 15 min at room temperature (RT) to separate serum from clotted blood. The serum was aliquoted and stored at −80°C.

### Determination of anti-CrpA and anti-AspA titers in patient serum

The 96-well ELISA plates (Nunc MaxiSorp, Thermo Fisher Scientific, Massachusetts, USA) were coated with recombinant CrpA or AspA protein (2 µg/mL) in 1 x ELISA coating buffer (Biolegend, California, USA) at 4°C overnight. Afterward, plates were blocked with 2.5% bovine serum albumin (BSA)/2.5% skimmed milk powder (SMP; Carl Roth GmbH, Germany) in PBS and 0.05% Tween 20 (neoFroxx GmbH, Germany; PBST) for 120 min at RT. Serum was added in serial dilutions in a blocking buffer for 120 min at RT. Plates were then incubated with horseradish peroxidase (HPR)-conjugated goat anti-human IgG antibody (Jackson ImmunoResearch, Pennsylvania, USA) diluted 1:2,500 in blocking buffer for 60 min at RT. Between each step, plates were washed several times with PBST. ELISAs were developed with ABTS solution (Thermo Fisher Scientific, Massachusetts, USA), and absorbance was measured at 415 nm.

### Immunofluorescence

Samples were analyzed using a Zeiss LSM 780 laser scanning confocal microscope (Carl Zeiss Ltd) using Zen software (Carl Zeiss Ltd) and a STELLARIS 5 Confocal Microscope (Leica Microsystems) equipped with HyD detectors and operated with LAS X software. For immunofluorescence confocal microscopy, *A. fumigatus* conidia were produced in MM. Plates were washed with sterile PBS, followed by centrifugation and filtration through sterile Miracloth (Millipore, 475855) to obtain conidia suspensions. Conidia concentration was determined by light microscopy using a Neubauer chamber. To ensure fungal cell adherence during staining procedures, 13  mm glass round coverslips were pretreated with BioBond (EMS, Hatfield, PA, USA) according to the manufacturer’s instructions; 2.5 × 10^5^ BMDM were seeded onto a coverslip in DMEM (placed at the bottom of the well of a 24-well plate) for 16 h. Next, BMDMs were stimulated with *A. fumigatus* conidia at a 10:1 MOI. Plates containing infected BMDM or conidia were centrifuged at 1,500 × *g* for 5 min and then incubated for 30 min at 4°C, rinsed with warm media, and incubated at 37°C with 5% of CO_2_. After 4 h, samples were then rinsed twice with PBS and fixed in 4% paraformaldehyde (wt/vol; Sigma Millipore, P6148) in PBS for 20 min. All the steps from now on were performed at room temperature. After washing twice in PBS, samples were quenched with 0.1 M glycine (Millipore Sigma, G7126) in PBS for 5 min. Samples were permeabilized with 0.05% Triton X-100 (wt/vol; Millipore Sigma) for 10  min. Next, samples were rinsed thoroughly in PBS and blocked with 2% BSA and 5  mg/mL normal donkey IgG (Jackson ImmunoResearch Laboratories Inc., 017-000-003) in PBS for 1 h. Samples were labeled with the primary antibody diluted in PBS containing 1% BSA (wt/vol) for 1  h. The samples were thoroughly rinsed in PBS and incubated for 30  min with secondary antibody, goat IgG anti-Rabbit IgG conjugated with Alexa Fluor-594, diluted 1:750 in PBS. For conidia wall and cellular membrane staining, samples were incubated for 5 min with Wheat Germ Agglutinin (WGA)-TRITC in PBS (Thermo Fisher Scientific; W7024, IF 1:300). Next, samples were rinsed in PBS, then in ddH_2_O, and mounted onto microscope slides with Fluoromount-G (Invitrogen, 00-4958-02). Samples incubated without the primary antibody served as controls and were all negative. Samples were analyzed using a Zeiss LSM 780 laser scanning confocal microscope (Carl Zeiss Ltd) using Zen software (Carl Zeiss Ltd). ImageJ software (57) was used to analyze and reconstruct the confocal images. Rabbit polyclonal antibody anti-GFP-Alexa Fluor 594 (Thermo Fisher Scientific, A21312, IF: 1:300) and Alexa Fluor 594 polyclonal goat anti-rabbit IgG (Thermo Fisher Scientific, A11012, IF: 1:750), and Hoechst 33342 Stain (H3570, ThermoFisher Scientific) were used in the experiments.

### Staining and labeling of cell surface components

Cell wall surface polysaccharide staining was performed as described previously (58). Briefly, 10^4^ spores for each deletion mutant and A1163 strains were inoculated in 200 µL of liquid MM and incubated for 4 h at 37°C (swollen conidia) and 4°C (resting conidia) before the culture medium was removed and conidia were UV-irradiated (600,000 mJ). For dectin labeling, 200 µL of a blocking solution (2% [vol/vol] goat serum, 1% [vol/vol] bovine serum albumin [BSA], 0.1% [vol/vol] Triton X-100, 0.05% [vol/vol] Tween 20, 0.05% [vol/vol] sodium azide, and 0.01 M PBS) was added to each well. Samples were incubated for 30 min at room temperature (RT), and 0.2 µg/mL of Fc-h-dectin-hFc (Invivogen) was added to the UV-irradiated conidia and incubated for 1 h at RT, followed by the addition of 1:1,000 DyLight 594-conjugated goat anti-human IgG1 (Abcam) for 1 h at RT. Conidia were then washed with phosphate-buffered saline (PBS), and fluorescence was read at 587 nm excitation and 615 nm emission. For chitin staining, 200 µL of a PBS solution with 10 mg/mL of calcofluor white (CFW) was added to the UV-irradiated conidia, which were incubated for 5 min at RT and washed with PBS before fluorescence was read at 380 nm excitation and 450 nm emission. All experiments were performed using at least four repetitions, and fluorescence was read in a microtiter plate reader (Synergy HTX Multimode Reader; Agilent Biotek or EnSpire Multimode Plate Reader; PerkinElmer).

### Statistical analysis

Grouped column plots with standard deviation error bars were used for representations of data. For comparisons with data from wild-type or control conditions, we performed paired t tests, one-way analysis of variance (ANOVA), or two-way analysis of variance (ANOVA). All statistical analyses and graphics building were performed by using GraphPad Prism 9 (GraphPad Prism Software).

## Data Availability

The datasets generated for this study are available on request to the corresponding author.

## References

[1] van de Veerdonk FL, Gresnigt MS, Romani L, Netea MG, Latgé J-P. 2017. Aspergillus fumigatus morphology and dynamic host interactions. Nat Rev Microbiol 15:661–674. doi:10.1038/nrmicro.2017.90 28919635

[2] van de Veerdonk FL, Carvalho A, Wauters J, Chamilos G, Verweij PE. 2025. Aspergillus fumigatus biology, immunopathogenicity and drug resistance. Nat Rev Microbiol 2. doi:10.1038/s41579-025-01180-z 40316713

[3] Latgé JP, Chamilos G. 2019. Aspergillus fumigatus and aspergillosis in 2019. Clin Microbiol Rev 33:e00140-18. doi:10.1128/CMR.00140-18 31722890 PMC6860006

[4] Bayry J, Beaussart A, Dufrêne YF, Sharma M, Bansal K, Kniemeyer O, Aimanianda V, Brakhage AA, Kaveri SV, Kwon-Chung KJ, Latgé J-P, Beauvais A. 2014. Surface structure characterization of Aspergillus fumigatus conidia mutated in the melanin synthesis pathway and their human cellular immune response. Infect Immun 82:3141–3153. doi:10.1128/IAI.01726-14 24818666 PMC4136205

[5] Heinekamp T, Schmidt H, Lapp K, Pähtz V, Shopova I, Köster-Eiserfunke N, Krüger T, Kniemeyer O, Brakhage AA. 2015. Interference of Aspergillus fumigatus with the immune response. Semin Immunopathol 37:141–152. doi:10.1007/s00281-014-0465-1 25404120 PMC4326658

[6] Blango MG, Pschibul A, Rivieccio F, Krüger T, Rafiq M, Jia L-J, Zheng T, Goldmann M, Voltersen V, Li J, Panagiotou G, Kniemeyer O, Brakhage AA. 2020. Dynamic surface proteomes of allergenic fungal conidia. J Proteome Res 19:2092–2104. doi:10.1021/acs.jproteome.0c00013 32233371

[7] Aimanianda V, Bayry J, Bozza S, Kniemeyer O, Perruccio K, Elluru SR, Clavaud C, Paris S, Brakhage AA, Kaveri SV, Romani L, Latgé J-P. 2009. Surface hydrophobin prevents immune recognition of airborne fungal spores. Nature 460:1117–1121. doi:10.1038/nature08264 19713928

[8] Valsecchi I, Dupres V, Stephen-Victor E, Guijarro JI, Gibbons J, Beau R, Bayry J, Coppee J-Y, Lafont F, Latgé J-P, Beauvais A. 2017. Role of hydrophobins in Aspergillus fumigatus. J Fungi (Basel) 4:2. doi:10.3390/jof4010002 29371496 PMC5872305

[9] Voltersen V, Blango MG, Herrmann S, Schmidt F, Heinekamp T, Strassburger M, Krüger T, Bacher P, Lother J, Weiss E, Hünniger K, Liu H, Hortschansky P, Scheffold A, Löffler J, Krappmann S, Nietzsche S, Kurzai O, Einsele H, Kniemeyer O, Filler SG, Reichard U, Brakhage AA. 2018. Proteome analysis reveals the conidial surface protein CcpA essential for virulence of the pathogenic fungus Aspergillus fumigatus mBio 9:e01557-18. doi:10.1128/mBio.01557-18 30279286 PMC6168859

[10] Azimova D, Herrera N, Duvenage L, Voorhies M, Rodriguez RA, English BC, Hoving JC, Rosenberg O, Sil A. 2022. Cbp1, a fungal virulence factor under positive selection, forms an effector complex that drives macrophage lysis. PLoS Pathog 18:e1010417. doi:10.1371/journal.ppat.1010417 35731824 PMC9255746

[11] Dang EV, Lei S, Radkov A, Volk RF, Zaro BW, Madhani HD. 2022. Secreted fungal virulence effector triggers allergic inflammation via TLR4. Nature 608:161–167. doi:10.1038/s41586-022-05005-4 35896747 PMC9744105

[12] Basso P, Dang EV, Urisman A, Cowen LE, Madhani HD, Noble SM. 2022. Deep tissue infection by an invasive human fungal pathogen requires lipid-based suppression of the IL-17 response. Cell Host Microbe 30:1589–1601. doi:10.1016/j.chom.2022.10.004 36323314 PMC9744107

[13] Jia L-J, Rafiq M, Radosa L, Hortschansky P, Cunha C, Cseresnyés Z, Krüger T, Schmidt F, Heinekamp T, Straßburger M, Löffler B, Doenst T, Lacerda JF, Campos A Jr, Figge MT, Carvalho A, Kniemeyer O, Brakhage AA. 2023. Aspergillus fumigatus hijacks human p11 to redirect fungal-containing phagosomes to non-degradative pathway. Cell Host Microbe 31:373–388. doi:10.1016/j.chom.2023.02.002 36893734 PMC10016320

[14] Pinzan CF, Valero C, de Castro PA, da Silva JL, Earle K, Liu H, Horta MAC, Kniemeyer O, Krüger T, Pschibul A, et al.. 2024. Aspergillus fumigatus conidial surface-associated proteome reveals factors for fungal evasion and host immunity modulation. Nat Microbiol 9:2710–2726. doi:10.1038/s41564-024-01782-y 39191887 PMC11699518

[15] Boucher MJ, Madhani HD. 2024. Convergent evolution of innate immune-modulating effectors in invasive fungal pathogens. Trends Microbiol 32:435–447. doi:10.1016/j.tim.2023.10.011 37985333

[16] Peters-Golden M, Coffey M. 1999. Role of leukotrienes in antimicrobial host defense of the lung. Clin Rev Allergy Immunol 17:261–269. doi:10.1007/BF02737609 10436871

[17] Tilley SL, Coffman TM, Koller BH. 2001. Mixed messages: modulation of inflammation and immune responses by prostaglandins and thromboxanes. J Clin Invest 108:15–23. doi:10.1172/JCI13416 11435451 PMC209346

[18] Sheppe AEF, Edelmann MJ. 2021. Roles of eicosanoids in regulating inflammation and neutrophil migration as an innate host response to bacterial infections. Infect Immun 89:e0009521. doi:10.1128/IAI.00095-21 34031130 PMC8281227

[19] Harris SG, Padilla J, Koumas L, Ray D, Phipps RP. 2002. Prostaglandins as modulators of immunity. Trends Immunol 23:144–150. doi:10.1016/s1471-4906(01)02154-8 11864843

[20] Pettipher R, Hansel TT, Armer R. 2007. Antagonism of the prostaglandin D2 receptors DP1 and CRTH2 as an approach to treat allergic diseases. Nat Rev Drug Discov 6:313–325. doi:10.1038/nrd2266 17396136

[21] Claar D, Hartert TV, Peebles RS Jr. 2015. The role of prostaglandins in allergic lung inflammation and asthma. Expert Rev Respir Med 9:55–72. doi:10.1586/17476348.2015.992783 25541289 PMC4380345

[22] Zeng C, Liu J, Zheng X, Hu X, He Y. 2023. Prostaglandin and prostaglandin receptors: present and future promising therapeutic targets for pulmonary arterial hypertension. Respir Res 24:263. doi:10.1186/s12931-023-02559-3 37915044 PMC10619262

[23] Lee K, Lee SH, Kim TH. 2020. The biology of prostaglandins and their role as a target for allergic airway disease therapy. Int J Mol Sci 21:1851. doi:10.3390/ijms21051851 32182661 PMC7084947

[24] Chen S, Saeed AFUH, Liu Q, Jiang Q, Xu H, Xiao GG, Rao L, Duo Y. 2023. Macrophages in immunoregulation and therapeutics. Signal Transduct Target Ther 8:207. doi:10.1038/s41392-023-01452-1 37211559 PMC10200802

[25] Saleh LS, Vanderheyden C, Frederickson A, Bryant SJ. 2020. Prostaglandin E2 and its receptor EP2 modulate macrophage activation and fusion in vitro ACS Biomater Sci Eng 6:2668–2681. doi:10.1021/acsbiomaterials.9b01180 33463295

[26] Thrikawala S, Niu M, Keller NP, Rosowski EE. 2022. Cyclooxygenase production of PGE2 promotes phagocyte control of A. fumigatus hyphal growth in larval zebrafish. PLoS Pathog 18:e1010040. doi:10.1371/journal.ppat.1010040 35333905 PMC8986117

[27] Lamon G, Lends A, Valsecchi I, Wong SSW, Duprès V, Lafont F, Tolchard J, Schmitt C, Mallet A, Grélard A, Morvan E, Dufourc EJ, Habenstein B, Guijarro JI, Aimanianda V, Loquet A. 2023. Solid-state NMR molecular snapshots of Aspergillus fumigatus cell wall architecture during a conidial morphotype transition. Proc Natl Acad Sci USA 120:e2212003120. doi:10.1073/pnas.2212003120 36719915 PMC9963690

[28] Ghassemi N, Poulhazan A, Deligey F, Mentink-Vigier F, Marcotte I, Wang T. 2022. Solid-state NMR investigations of extracellular matrixes and cell walls of algae, bacteria, fungi, and plants. Chem Rev 122:10036–10086. doi:10.1021/acs.chemrev.1c00669 34878762 PMC9486976

[29] Delcourte L, Berbon M, Rodriguez M, Subban K, Lends A, Grélard A, Morvan E, Habenstein B, Saupe SJ, Delhaes L, Aimanianda V, Daskalov A, Loquet A. 2024. Magic-angle spinning NMR spectral editing of polysaccharides in whole cells using the DREAM scheme. Methods 230:59–67. doi:10.1016/j.ymeth.2024.07.003 39047926

[30] Gautam I, Singh K, Dickwella Widanage MC, Yarava JR, Wang T. 2024. New vision of cell walls in Aspergillus fumigatus from solid-state NMR spectroscopy. J Fungi (Basel) 10:219. doi:10.3390/jof10030219 38535227 PMC10971067

[31] Kang X, Kirui A, Muszyński A, Widanage MCD, Chen A, Azadi P, Wang P, Mentink-Vigier F, Wang T. 2018. Molecular architecture of fungal cell walls revealed by solid-state NMR. Nat Commun 9:2747. doi:10.1038/s41467-018-05199-0 30013106 PMC6048167

[32] Chakraborty A, Fernando LD, Fang W, Dickwella Widanage MC, Wei P, Jin C, Fontaine T, Latgé J-P, Wang T. 2021. A molecular vision of fungal cell wall organization by functional genomics and solid-state NMR. Nat Commun 12:6346. doi:10.1038/s41467-021-26749-z 34732740 PMC8566572

[33] Kupfahl C, Tsikas D, Niemann J, Geginat G, Hof H. 2012. Production of prostaglandins, isoprostanes and thromboxane by Aspergillus fumigatus: identification by gas chromatography-tandem mass spectrometry and quantification by enzyme immunoassay. Mol Immunol 49:621–627. doi:10.1016/j.molimm.2011.10.010 22118804

[34] Wang Y, Liu T, Gong H, Zhou Q, Wang Y, Sun S, Xie L. 2007. Gene profiling in murine corneas challenged with Aspergillus fumigatus. Mol Vis 13:1226–1233. 17679939

[35] Mezger M, Kneitz S, Wozniok I, Kurzai O, Einsele H, Loeffler J. 2008. Proinflammatory response of immature human dendritic cells is mediated by dectin-1 after exposure to Aspergillus fumigatus germ tubes. J Infect Dis 197:924–931. doi:10.1086/528694 18279049

[36] Liu H, Zheng M, Qiao J, Dang Y, Zhang P, Jin X. 2014. Role of prostaglandin D2 /CRTH2 pathway on asthma exacerbation induced by Aspergillus fumigatus. Immunology 142:78–88. doi:10.1111/imm.12234 24329550 PMC3992050

[37] Günther K, Nischang V, Cseresnyés Z, Krüger T, Sheta D, Abboud Z, Heinekamp T, Werner M, Kniemeyer O, Beilhack A, Figge MT, Brakhage AA, Werz O, Jordan PM. 2024. Aspergillus fumigatus-derived gliotoxin impacts innate immune cell activation through modulating lipid mediator production in macrophages. Immunology 173:748–767. doi:10.1111/imm.13857 39268960

[38] Mochon AB, Jin Y, Kayala MA, Wingard JR, Clancy CJ, Nguyen MH, Felgner P, Baldi P, Liu H. 2010. Serological profiling of a Candida albicans protein microarray reveals permanent host-pathogen interplay and stage-specific responses during candidemia. PLoS Pathog 6:e1000827. doi:10.1371/journal.ppat.1000827 20361054 PMC2845659

[39] Käfer E. 1977. Meiotic and mitotic recombination in Aspergillus and its chromosomal aberrations. Adv Genet 19:33–131. doi:10.1016/s0065-2660(08)60245-x 327767

[40] Chaveroche M-K. 2000. A rapid method for efficient gene replacement in the filamentous fungus Aspergillus nidulans. Nucleic Acids Res 28:97e–997. doi:10.1093/nar/28.22.e97 11071951 PMC113889

[41] Colot HV, Park G, Turner GE, Ringelberg C, Crew CM, Litvinkova L, Weiss RL, Borkovich KA, Dunlap JC. 2006. A high-throughput gene knockout procedure for Neurospora reveals functions for multiple transcription factors. Proc Natl Acad Sci USA 103:10352–10357. doi:10.1073/pnas.0601456103 16801547 PMC1482798

[42] Marim FM, Silveira TN, Lima DS Jr, Zamboni DS. 2010. A method for generation of bone marrow-derived macrophages from cryopreserved mouse bone marrow cells. PLoS One 5:e15263. doi:10.1371/journal.pone.0015263 21179419 PMC3003694

[43] Strober W. 2015. Trypan blue exclusion test of cell viability. Curr Protoc Immunol 111:A3. doi:10.1002/0471142735.ima03bs111 PMC6716531 26529666

[44] Deng Q, Yoo SK, Cavnar PJ, Green JM, Huttenlocher A. 2011. Dual roles for Rac2 in neutrophil motility and active retention in zebrafish hematopoietic tissue. Dev Cell 21:735–745. doi:10.1016/j.devcel.2011.07.013 22014524 PMC3199325

[45] Glass E, Robinson SL, Rosowski EE. 2025. Zebrafish use conserved CLR and TLR signaling pathways to respond to fungal PAMPs in zymosan. Dev Comp Immunol 162:105286. doi:10.1016/j.dci.2024.105286 39536806 PMC11740225

[46] Thrikawala S, Rosowski EE. 2020. Infection of zebrafish larvae with Aspergillus spores for analysis of host-pathogen interactions. J Vis Exp 16. doi:10.3791/61165 PMC9435317 32478760

[47] Sorgi CA, Peti APF, Petta T, Meirelles AFG, Fontanari C, Moraes LAB de, Faccioli LH. 2018. Comprehensive high-resolution multiple-reaction monitoring mass spectrometry for targeted eicosanoid assays. Sci Data 5:180167. doi:10.1038/sdata.2018.167 30129930 PMC6103261

[48] Böckmann A, Gardiennet C, Verel R, Hunkeler A, Loquet A, Pintacuda G, Emsley L, Meier BH, Lesage A. 2009. Characterization of different water pools in solid-state NMR protein samples. J Biomol NMR 45:319–327. doi:10.1007/s10858-009-9374-3 19779834

[49] Vranken WF, Boucher W, Stevens TJ, Fogh RH, Pajon A, Llinas M, Ulrich EL, Markley JL, Ionides J, Laue ED. 2005. The CCPN data model for NMR spectroscopy: development of a software pipeline. Proteins 59:687–696. doi:10.1002/prot.20449 15815974

[50] Johnson M, Zaretskaya I, Raytselis Y, Merezhuk Y, McGinnis S, Madden TL. 2008. NCBI BLAST: a better web interface. Nucleic Acids Res 36:W5–W9. doi:10.1093/nar/gkn201 18440982 PMC2447716

[51] Sayers EW, Bolton EE, Brister JR, Canese K, Chan J, Comeau DC, Connor R, Funk K, Kelly C, Kim S, Madej T, Marchler-Bauer A, Lanczycki C, Lathrop S, Lu Z, Thibaud-Nissen F, Murphy T, Phan L, Skripchenko Y, Tse T, Wang J, Williams R, Trawick BW, Pruitt KD, Sherry ST. 2022. Database resources of the national center for biotechnology information. Nucleic Acids Res 50:D20–D26. doi:10.1093/nar/gkab1112 34850941 PMC8728269

[52] Katoh K, Rozewicki J, Yamada KD. 2019. MAFFT online service: multiple sequence alignment, interactive sequence choice and visualization. Brief Bioinform 20:1160–1166. doi:10.1093/bib/bbx108 28968734 PMC6781576

[53] Nguyen L-T, Schmidt HA, von Haeseler A, Minh BQ. 2015. IQ-TREE: a fast and effective stochastic algorithm for estimating maximum-likelihood phylogenies. Mol Biol Evol 32:268–274. doi:10.1093/molbev/msu300 25371430 PMC4271533

[54] Letunic I, Bork P. 2021. Interactive Tree Of Life (iTOL) v5: an online tool for phylogenetic tree display and annotation. Nucleic Acids Res 49:W293–W296. doi:10.1093/nar/gkab301 33885785 PMC8265157

[55] Denning DW, Cadranel J, Beigelman-Aubry C, Ader F, Chakrabarti A, Blot S, Ullmann AJ, Dimopoulos G, Lange C, European Society for Clinical Microbiology and Infectious Diseases and European Respiratory Society. 2016. Chronic pulmonary aspergillosis: rationale and clinical guidelines for diagnosis and management. Eur Respir J 47:45–68. doi:10.1183/13993003.00583-2015 26699723

[56] Donnelly JP, Chen SC, Kauffman CA, Steinbach WJ, Baddley JW, Verweij PE, Clancy CJ, Wingard JR, Lockhart SR, Groll AH, et al.. 2020. Revision and update of the consensus definitions of invasive fungal disease from the European Organization for Research and Treatment of Cancer and the Mycoses Study Group Education and Research Consortium. Clin Infect Dis 71:1367–1376. doi:10.1093/cid/ciz1008 31802125 PMC7486838

[57] Schindelin J, Arganda-Carreras I, Frise E, Kaynig V, Longair M, Pietzsch T, Preibisch S, Rueden C, Saalfeld S, Schmid B, Tinevez J-Y, White DJ, Hartenstein V, Eliceiri K, Tomancak P, Cardona A. 2012. Fiji: an open-source platform for biological-image analysis. Nat Methods 9:676–682. doi:10.1038/nmeth.2019 22743772 PMC3855844

[58] Winkelströter LK, Bom VLP, de Castro PA, Ramalho LNZ, Goldman MHS, Brown NA, Rajendran R, Ramage G, Bovier E, Dos Reis TF, Savoldi M, Hagiwara D, Goldman GH. 2015. High osmolarity glycerol response PtcB phosphatase is important for Aspergillus fumigatus virulence. Mol Microbiol 96:42–54. doi:10.1111/mmi.12919 25597841

